# Exosome-transmitted circCABIN1 promotes temozolomide resistance in glioblastoma via sustaining ErbB downstream signaling

**DOI:** 10.1186/s12951-023-01801-w

**Published:** 2023-02-08

**Authors:** Xiao Liu, Qingdong Guo, Guangxun Gao, Zhengcong Cao, Zhihao Guan, Bo Jia, Weizhong Wang, Kuo Zhang, Wangqian Zhang, Shuning Wang, Weina Li, Qiang Hao, Yingqi Zhang, Meng Li, Wei Zhang, Jintao Gu

**Affiliations:** 1 State Key Laboratory of Cancer Biology, School of Pharmacy, Biotechnology Center, The Fourth Military Medical University, Xi’an, China; 2 Department of Hematology, Xijing Hospital, Xi’an, China; 3 Department of Neurosurgery, Xijing Hospital, Xi’an, China

**Keywords:** Glioblastoma, Temozolomide resistance, Exosome, circCABIN1, Stemness

## Abstract

Although temozolomide (TMZ) provides significant clinical benefit for glioblastoma (GBM), responses are limited by the emergence of acquired resistance. Here, we demonstrate that exosomal circCABIN1 secreted from TMZ-resistant cells was packaged into exosomes and then disseminated TMZ resistance of receipt cells. CircCABIN1 could be cyclized by eukaryotic translation initiation factor 4A3 (EIF4A3) and is highly expressed in GBM tissues and glioma stem cells (GSCs). CircCABIN1 is required for the self-renewal maintenance of GSCs to initiate acquired resistance. Mechanistically, circCABIN1 regulated the expression of olfactomedin-like 3 (OLFML3) by sponging miR-637. Moreover, upregulation of OLFML3 activating the ErbB signaling pathway and ultimately contributing to stemness reprogramming and TMZ resistance. Treatment of GBM orthotopic mice xenografts with engineered exosomes targeting circCABIN1 and OLFML3 provided prominent targetability and had significantly improved antitumor activity of TMZ. In summary, our work proposed a novel mechanism for drug resistance transmission in GBM and provided evidence that engineered exosomes are a promising clinical tool for cancer prevention and therapy.

**Graphical Abstract:**

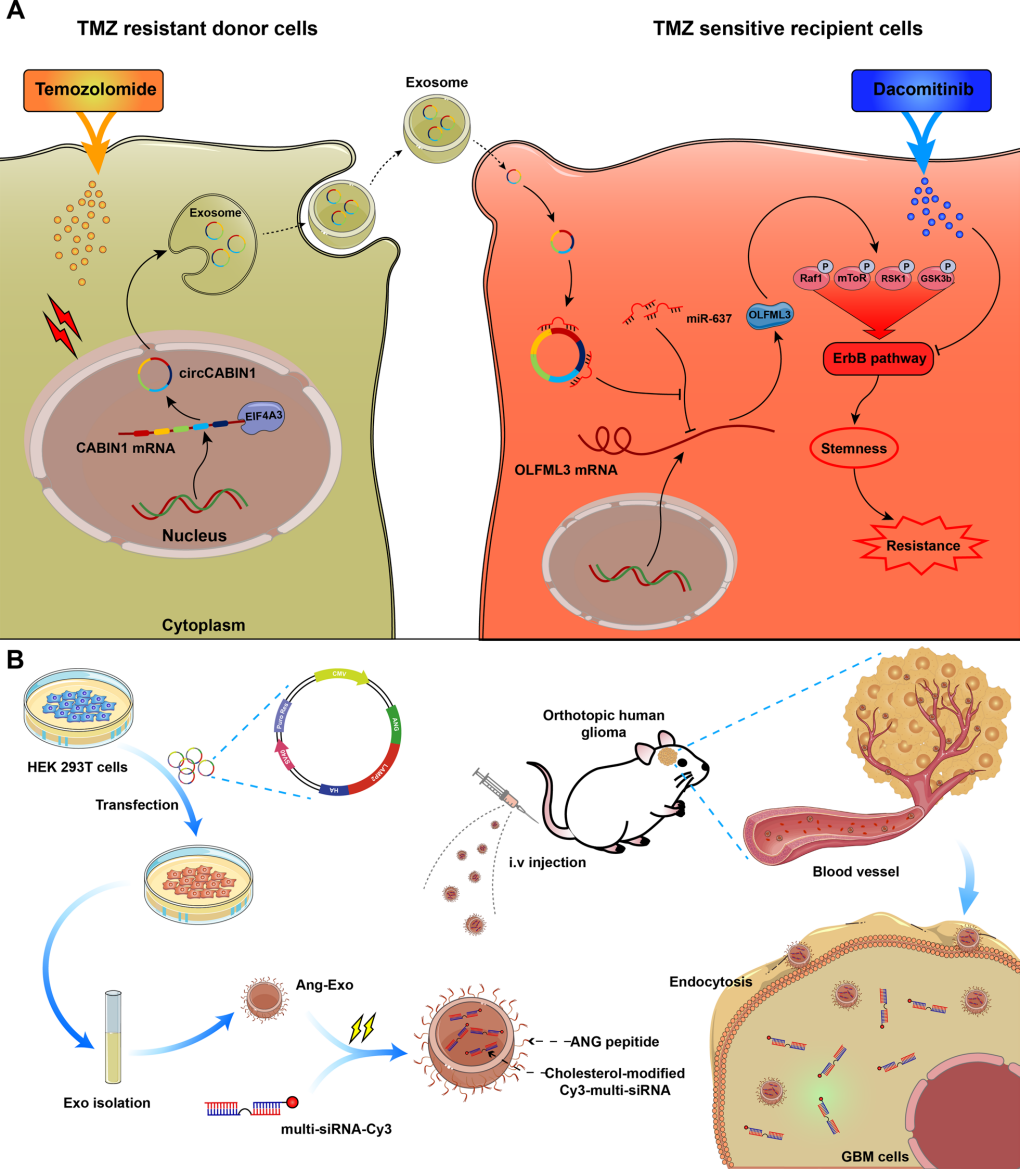

**Supplementary Information:**

The online version contains supplementary material available at 10.1186/s12951-023-01801-w.

## Introduction

Glioblastoma (GBM) represents the most common and fatal form of malignant primary glioma and has a median overall survival of approximately 14.6 months after diagnosis [1]. Even if active surgery is combined with radiotherapy and chemotherapy, the treatment effect in GBM is poor [2]. Temozolomide (TMZ), a second-generation oral alkylating agent, can readily pass through the blood–brain barrier (BBB) and is currently used as a first-line therapy for GBM [3]. However, major obstacles to effective treatment are postoperative tumor recurrence and acquired resistance to TMZ [4]. Currently, patients with advanced GBM who develop resistance to TMZ have limited clinical therapeutic options. Hence, it is necessary to investigate the biological basis of TMZ resistance and identify novel targets for preventing and overcoming TMZ resistance.

Two main biological characteristics of GBM are its high recurrence and heterogeneity [5]. More recently, it has been shown that multiple subtypes coexist in different regions and different cells within the same tumor [6]. This interpatient and intratumoral heterogeneity poses a daunting challenge for research programs aimed at developing targeted therapeutic approaches and may explain the failures of these approaches for GBM. Multiple ways may be involved in the transduction of signals among different cells within the same tumor. One possible mechanism is exosome-mediated intercellular transport, which allows cells to send and receive molecules that otherwise could not be transferred, including proteins, circular RNAs (circRNAs), long noncoding RNAs (lncRNAs) and microRNAs (miRNAs) [7, 8]. Exosomes, an efficient intercellular signal delivery system working at both proximal and distal sites, have recently been identified to play a key role in cancer development [9]. A recent study showed that chemotherapy-elicited exosomes promoted mammary tumor metastasis [10]. Exosomes derived from highly metastatic melanomas increased the malignancy of low metastatic melanomas by educating bone marrow progenitor cells [11]. Marat S et al. [12] reported that apoptotic cell-derived exosomes promote glioblastoma malignancy via intercellular transfer of splicing factors. However, whether exosomes derived from resistant cancer cells can confer drug resistance on sensitive cells needs elucidation.

Recently, circRNAs have been shown to be definitively enriched in exosomes and are easy to measure in the circulation; in addition, with their covalently closed loop structures without 5′ to 3′ polarity or polyadenylated tails, they are highly stable compared with their parental linear RNAs [13–15]. Moreover, compared with linear RNAs, circRNAs are more easily sorted into exosomes [16]. CircRNAs show disease-specific and disease-progression specific characteristics and exhibit potent biological functions in regulating malignant behaviors such as proliferation, migration, autophagy, and drug resistance [17–19]. However, whether circRNAs cause TMZ resistance in GBM cells and can transmit this resistance to sensitive recipient cells by being packaged into exosomes is not well known.

In this study, we investigated the hypothesis that exosomes secreted by TMZ-resistant GBM cells cause a phenotypic shift in sensitive recipient tumor cells to promote their chemoresistance due to changes mediated by exosomal circRNAs. Exosomal circRNAs conferred a resistance phenotype on recipient GBM cells by modulating the cancer stemness signature. Moreover, we designed a functionalized exosome delivery system that can efficiently deliver multiplex small interfering RNA (multi-siRNA) for targeted therapy of GBM. Therefore, this study identifies a novel network mediated by exosomal circRNAs that regulates the chemoresistance of GBM cells and provides novel approaches to enhance their chemosensitivity.

## Materials and methods

### Cell culture

Human GBM cell lines LN229 was obtained from ATCC and cultured in Dulbecco’s modified Eagle’s medium (Gibco, Shanghai, China) containing 10% fetal bovine serum (FBS; Gibco), 1% penicillin/streptomycin (NCM, Suzhou, China). For patient-derived GBM cell culture (GBM 02, GBM 04 and GBM 15), fresh brain GBM tissues were collected within 30 min after tumor resection, washed, minced, and enzymatically dissociated. The resulting cell suspension was then strained through a 70 μm cell strainer (Falcon). The single suspension of cells was washed in Phosphate Buffer Saline (PBS) twice. Adherent GBM cells were resuspended and cultured in DMEM/F12 medium containing 10% FBS and 1% penicillin/streptomycin. GSC were cultured in in UltraLow attachment plates in GSC medium that contained Neurobasal A media supplemented with B27, heparin (5 μg/ml, Sigma-Aldrich), epidermal growth factor (EGF, 20 ng/ml) and basic fibroblast growth factor (bFGF, 20 ng/ml, Peprotech), bFGF and EGF were added twice a week and the cultural medium was changed every 7 days. All cells are tested for mycoplasma contamination each month. All cells used in this paper are mycoplasma free. Acquisition and use of these tissues were approved by the Ethics Committee of Fourth Military Medical University.

### Transfection and infection

siRNAs, plasmid and cholesterol-modified cy3-multi-siRNA were designed and synthesized by Tsingke Biotechnology Co., Ltd. (Beijing, China), and sequences of siRNAs were shown in supplementary materials. The plasmids pcDNA3.1( +)-ANG-LAMP2B-HA were constructed by our laboratory and verified by OBiO Technology Corp., Ltd. (Shanghai, China). Cells were transfected with plasmids or siRNA by Lipofectamine 3000 (Invitrogen, CA, USA) according to the manufacturer’s instructions.

### RNA isolation, reverse transcription, and qRT-PCR

Total RNA was isolated from cell lines and clinical samples using TRIzol reagent (Invitrogen) according to the manufacturer’s instructions. In addition, nuclear and cytoplasmic RNA was extracted using a PARIS™ Kit (Thermo Scientific, USA). Reverse transcription was performed to synthesize cDNA using the the Prime Script RT Reagent Kit (TaKaRa). Real-time PCR analyses were performed using Fast SYBR® Green Master Mix (Thermo Scientific, USA). The relative expression levels of circRNA and mRNA were calculated using the 2^−ΔΔCT^ method on an Applied Biosystems®7500 Fast instrument (ThermoFisher, USA). GAPDH was used as an internal control to normalize the expression of target genes. Primer sequences are listed in Additional file 1.

### Actinomycin D (Act D), RNase R and subcellular fraction analysis

For Act D assay, LN229 cells were exposed to Actinomycin D (Sigma-Aldrich, St. Louis, MO, USA) for 4, 8, 12 and 24 h. The cells were harvested according to the time of treatment. For RNase R assay, LN229 cells and divided into two groups for control and RNase R treatment. The sample was incubated for 30 min at 37 ℃ with 2U/μg RNase R (Geneseed, Guangzhou, China). After the above treatments, the abundance of circCABIN1 or CABIN1 was determined via qRT-PCR.

### Western blotting and sliver stain

Total protein was extracted from cells with Western & IP lysis buffer (NCM) and quantified using BCA protein assay kit (Thermo Fisher Scientific). Equal amounts of lysate protein were separated by SDS-PAGE and transferred onto PVDF membranes (EMD Millipore), then blocked with 5% skimmed milk for 1 h. Membranes were incubated with primary antibodies at 4 °C overnight, followed by incubation with secondary antibody. Finally, membranes were probed using the Tanon5200 imaging system (Tanon, Shanghai, China). As described above, after the sample is separated by SDS-PAGE, silver staining assay used M5 Protein Silver Stain Kit (Mei5bio) according to the manufacturer’s instructions. The final image was obtained by Gel Doc XR + (BIO-RAD, USA).

### Colony formation assay

The LN229 and GBM02 cells were collected and seeded into 6-well plates, each experimental group was inoculated with 1000 cells/well and incubated at 37 °C for 14 days. After the removal of the culture medium, cells were washed with PBS, fixed in 4% paraformaldehyde and stained with 0.1% crystal violet. Finally, the cells were photographed by camera and the number of colonies were then counted.

### Tumorsphere formation assay

Tumorsphere formation was analyzed as described previously. Briefly, cells were plated in 96-well ultralow attachment plates at a density of 1,000 cells per well and tumoursphere numbers and sizes were calculated at the seventh day after cell placement.

### Establishment of PDOs

PDOs were establishment and cultivation as described previously [20].

### In vitro limiting dilution assay

Limiting dilution assay was performed as we previously described [21]. In brief, dissociated cells from GBM spheres were seeded in 96-well plates containing GSCs culture medium. After 7 days, each well was examined for formation of tumor spheres. Stem cell frequency was calculated using extreme limiting dilution analysis (http://bioinf.wehi.edu.au/software/elda/).

### Immunofluorescence

The cells were washed twice with PBS, fixed with 4% paraformaldehyde for 15 min, and permeabilized with 0.5% Triton X-100 for 30 min at room temperature. Each step followed with PFS washed twice. The cells were incubated overnight at 4 °C with corresponding antibodies, after being blocked for 40 min with 3% BSA. Then, the cells were incubated for 1 h with fluorescent secondary antibody. The fluorescent was photographed under a confocal laser scanning microscope using Nikon NIS-Elements software (Nikon, Tokyo, Japan).

### RNA-fluorescence in situ hybridization (FISH)

The circCABIN1 and miR-637 FISH probes were designed and synthesized by GenePharma (Shanghai, China). To hybridize the probes to cells and tissues, the Fluorescent in Situ Hybridization Kit (RiboBio, China) was used following the manufacturer’s manual. All images were acquired on Nikon A1Si Laser Scanning confocal microscope.

### Caspase 3/7 assay

1 × 10^5^ cells were collected and seeded in 20 mm Glass Bottom Cell Culture Dish (NEST, China). After different treatment, Apo-ONE Homogeneous Caspase-3/7 asssy kit was used following the manufacturer’s manual. All images were acquired on Nikon A1Si Laser Scanning confocal microscope.

### Apoptosis assay

A FACSCalibur Flow Cytometer (BD Biosciences, USA) was used for the apoptosis assay followed the product manual. Cells were seeded in 6-well plates, harvesting in centrifuge tube after different treatment. Then washing, resuspension and staining were in accordance with the BD Pharmingen™ FITC Annexin V Apoptosis Detection Kit I (BD) protocol. The data were then analyzed with Flow Jo 10.0 software (Tree Star, San Francisco, CA, USA). Each assay was independently repeated three times.

### Histology and immunohistochemistry (IHC)

Tumor tissues were fixed in 4% paraformaldehyde and embedded in paraffin. Sections were then processed for H&E and IHC. Primary antibodies specific for Ki-67 (1: 1000, Abcam, USA), caspase 3 (1:100, Abcam, USA), EIF4A3 (1:200, Proteintech, China), OLFML3 (1:500, ThermoFisher Scientific, USA) and ALDH1A3(1: 200, Abcam, USA) were used. All images were recorded with an Olympus BX-51 light microscope.

### RNA immunoprecipitation (RIP)

RNA immunoprecipitation (RIP) assay was performed using Magna RIP ™ RNA-binding protein immunoprecipitation kit (Millipore, Billerica, MA, USA) following the manufacturer’s manual. LN229 cells were lysed in complete RNA immunoprecipitation lysis buffer. Afterwards, Ago2 or IgG antibody conjugated to magnetic beads was added to the cell extract incubating at 4 °C overnight. The beads were washed and incubated with Proteinase K to remove proteins. Finally, isolated RNA was extracted and purified for qRT-PCR analysis.

### Biotin-labeled RNA pull-down

RNA pull-down assay and was performed using Pierce™ Magnetic RNA–Protein Pull-Down Kit (Thermo Fisher Scientific) following the manufacturer’s manual. Upstream and downstream RNA fragments of circCABIN1 were synthesized by sangon biotech. Approximately 1 × 10^7^ cells were harvested and lysed. Then the lysate was incubated with labeled RNA and streptavidin magnetic beads at 4℃ for 1 h. Afterward, the magnetic beads were washed and the pull-down complexes were boiled with the loading buffer for western blot and slivers stain.

### Cell viability assay

For the cell viability assay, 5 × 10^3^ cells were plated in 96-well plates and treated in triplicate for 3 days with TMZ (TargetMol, USA). Cell proliferation was estimated using the Cell Counting Kit-8 (CCK-8, Abcam, USA) following the protocol, the absorbance was measured at 460 nm using a Microplate Reader (Biotek, USA).

### Luciferase reporter assay

For target gene detection, GBM cells were seeded at 1 × 10^4^ cells per well in a 96-well plate for 24 h before transfection. Cells were co-transfected with miR-637 mimics, miR-637 inhibitor, and psiCHECK2 constructs with WT or mutated target sequence. Twenty-four hours after transfection, the luciferase activity was measured using the Dual Luciferase Reporter Assay System (Promega).

### Protein array assay

LN229 cells were harvest after IgG or OLFML3 recombinant protein treatment. Total protein was extracted, quantified and administrated with RayBio C-Series Human and Mouse AKT Pathway Phosphorylation Array C1 following the protocol. Finally, the antibody arrays were probed using the Tanon5200 imaging system (Tanon, Shanghai, China).

### Exosome isolation and characterization

In order to prepare stable cell lines for modified exosome generation, pAng2-Lamp2b were transfected into the 293 T cells using Lipofectamine3000 according to the manufacturer's manual. After transfection, the medium was replaced with serum-free DMEM for 48 h and the medium was harvested. Then the obtained medium was centrifuged at 1500 g for 30 min to remove residual cells and debris. Exosomes were extracted from supernatant using Exosome Isolation Reagent (RiboBio). All of these steps were performed at 4 °C.

### Orthotopic mouse xenografts

Male, 6-week-old BALB/c nude mice (~ 18 g) were obtained from Gempharmatech Co., Ltd. (Nanjing, China). GBM cells expressing luciferase were intracranially transplanted into immunocompromised mice. In brief, a burr hole was made 2.5 mm left of the sagittal suture and 0.5 mm anterior to the bregma using a dental drill with a diameter of 0.7 mm, and the injection depth is 2.5 mm. To examine the tumor growth, animals were administrated intraperitoneally with 3.0 mg/100uL solution of D-Luciferin potassium salt (Abcam, USA) and anesthetized with isoflurane for the imaging analysis. The tumor luciferase images were captured by using an IVIS 100 imaging system (PerkinElmer, USA).

### Exosomes labeling

The fluorescent dye DiD was purchased from GOYOO (China) and used to label exosomes. Exosomes were incubated in the DiD working fluid (1:300, dilute with PBS) for 20 min at 37 ℃ then ultracentrifuged at 120,000 × g, 90 min to remove the unbounded dye. After being washed twice in PBS with 120,000 × *g* centrifugation, the labeled exosomes were resuspended in PBS prior to use.

### Animal studies

For in vivo evaluating delivery of engineered ANG-exo and unmod-exo (about 200 μg) were labeled with the DID and injected into the tumor-bearing mice via the tail vein to analyze the distribution of exosomes. Fluorescence signals were detected by IVIS at 0 h, 3 h, 6 h and 12 h after injection. Afterwards, the mice were sacrificed by cervical dislocation, then the organs and tumors were taken out, and the distribution of fluorescently labeled exosomes in various organs was observed using IVIS. All animal studies were performed in accordance with protocols approved by the Ethical Committee and Institutional Review Board of Fourth Military Medical University.

### In vivo safety evaluation

Eight female BALB/c mice were randomly divided into two groups. One group received an intravenous injection of ANG-multi-siRNA-exo (200 μg per mice) at one dose for 12 days and the other group was treated with Saline as control. Blood samples and major organ tissues were collected at 24 h after the last administration, for hematologic and histochemistry analysis. The serum alanine aminotransferase (ALT), aspartate aminotransferase (AST), creatinine (Cr) and blood urea nitrogen (BUN) levels were analyzed by Hitachi 7080 Chemistry Analyzer. Major organs such as brain, heart, lung, liver, spleen, and kidney were fixed with paraformaldehyde for 48 h and embedded in paraffin. All animal procedures were conducted in accordance with the care and use of laboratory animals’ protocol approved by the institutional animal care committee of FMMU.

### In vitro BBB model

An in-vitro BBB model was established using LN229 and bEnd.3 cells as described previously. In brief, bEnd.3 cells were seeded into the upper chamber of Transwell inserts coated with 2% gelatin solution and cultured for 7 days in DMEM supplemented with 10% FBS. LN229 cells were seeded in the lower Transwell compartment. unmod-multi-siRNA-exo and ANG-multi-siRNA-exo were added to the upper chamber to assess the penetration unmod-exo and ANG-exo efficiency across the BBB.

### Nanoparticle tracking analysis (NTA)

The NTA was performed as described previously. In brief, the size distribution of the exosomes were analyzed using a Nano Sight LM10 instrument (Marvern instruments, UK). The particle suspensions were diluted with PBS to a concentration of 108 particles/mL for analysis.

### Bioinformatics analyses of public GBM data set

The gene expression in human glioma and patient survival information were analyzed using gene-profiling data from the Gliovis database (http://gliovis.bioinfo.cnio.es/), Brainbase database (https://ngdc.cncb.ac.cn) and CGGA database (http://www.cgga.org.cn/).

### Statistical analysis

All the statistical analyses using GraphPad Prism 7.0 and SPSS 22.0. Data are presented as the mean ± standard deviation. Differences between the indicated groups were compared using the Student’s t-test and one-way analysis of variance (ANOVA) followed by Fisher’s least significant difference (LSD) test. Correlations were evaluated by Pearson correlation analysis. The log-rank test was used to evaluate significance in the cumulative overall survival (OS) rates calculated using the Kaplan–Meier method. For animal survival studies, blinding during analysis was used for all the in vivo experiments. Animals were randomly assigned to treatment group. A value of p < 0.05 was considered to be significant.

## Results

### Exosomes derived from TMZ-resistant cells disseminates drug resistance characteristics to sensitive cells

**Fig. 1:**
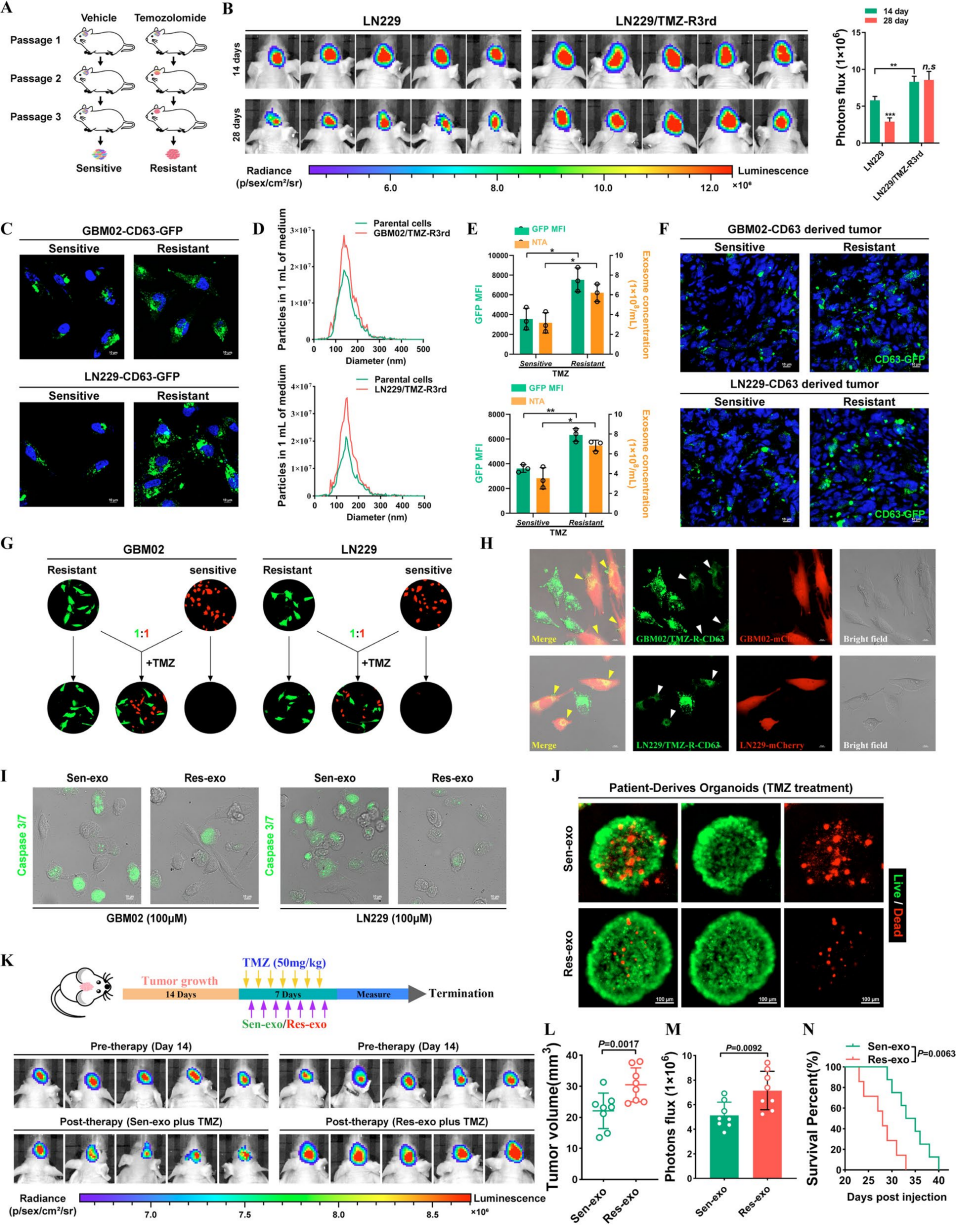
Temozolomide-resistant GBM cells transmit resistance to sensitive cells through exosomes. **A** Schematic model. Process to acquire Temozolomide-resistant cells. **B** Confirm the drug resistance of TMZ-resistant LN229 cells in vivo. Left, IVIS detects bioluminescence signals. Right, quantification of bioluminescent imaging signal intensities. **C** The expression of CD63 in TMZ-R cells and parental cells was detected by fusion expression of CD63-GFP protein. Nuclei were counterstained with DAPI. Scale bar, 10 μm. **D** Secretion of exosomes were detected by nanoparticle tracking analysis. **E** GFP mean fluorescence intensity (MFI; left y axis) and concentration by NTA (right y axis) of exosomes released by sensitive or resistant GBM cells. **F** Fluorescence images of Brain frozen section of brain from orthotopic GBM xenograft mice. Nuclei were counterstained with DAPI. Scale bar, 10 μm. **G** TMZ resistant cells (GFP) co-cultured with parental cells (mCherry) at a ratio of 1:1 with TMZ treatment (100uM) for 4 days. **H** TMZ-resistant cells expressed CD63-GFP fusion protein co-cultured with parental cells (mCherry) at a ratio of 1:1 for 4 days. Scale bar, 10 μm. **I** GBM cells were co-cultured with Res-exo or Sen-exo and TMZ (100 μM), and apoptosis was detected by caspase3/7 assay. Scale bar, 10 μm. **J** Patient-derived organoids was to confirm TMZ resistant after treated with Sen-exo / Res-exo. Scale bar, 50 μm. **K**–**N** Schematic model. Process of animal study. Nude mice were orthotopically xenografted with GBM cells (2 × 10^6^ cells) and treated intravenously with Res-exo or Sen-exo (200 μg per mouse) and intraperitoneally with TMZ (40 mg/kg) daily. IVIS detects bioluminescence signals. Quantification of tumor size (**L**), and quantification of bioluminescent imaging signal intensities (**M**), and survival rate (**N**) of mice in indicated groups are shown. Results are presented as mean ± SD. *p < 0.05, and **p < 0.01. NS, not significant

To generate TMZ-resistant GBM cells, we grafted GBM02 and LN229 cells into nude mice and performed cycles of TMZ treatment along with serial passage in these orthotopic models (Fig. 1A). GBM xenografts from the third passage exhibited a poor response to TMZ treatment. Resistant GBM cells were isolated from these xenografts and named LN229/TMZ-R3rd and GBM02/TMZ-R3rd cells. Compared with the corresponding parental cells, LN229/TMZ-R3rd and GBM02/TMZ-R3rd cells exhibited extremely poor responses to TMZ, as shown by the in vivo drug sensitivity test (Fig. 1B and Additional file 1: Fig. S1A). Exosomes are membrane-bound extracellular vesicles that are produced in the endosomal compartment of most eukaryotic cells. Intercellular communication via exosomes is a critical mediator of biological processes. Recently, accumulating studies have reported that exosomes perform important biological functions in humans, especially in the occurrence and development of tumors [10, 22].

We investigated whether TMZ can modulate exosome release from cancer cells. We then examined the effects of chemotherapy-TMZ on exosome release from cancer cells. To trace exosomes, LN229 and GBM02 cells were modified to express a fluorescent CD63-GFP fusion protein targeted to exosomal membranes (Fig. 1C). Nanoparticle tracking analysis (NTA) of conditioned medium demonstrated that TMZ-resistant cells released a significantly higher number of exosomes than TMZ-sensitive cells. In addition, CD63-GFP fluorescence was increased in TMZ-resistant GBM cells (Fig. 1D, E). After cycles of TMZ treatment along with serial passage in these orthotopic models, we found that GFP fluorescence was increased in resistant tumors compared with the parental tumors (Fig. 1F). However, TMZ treatment did not alter the physical features of resistant cell-derived exosomes, as demonstrated by transmission electron microscopy (Additional file 1: Fig. S1B).

Exosomes affect tumor resistance to various drugs, prompting us to explore whether TMZ-resistant cell-derived exosomes alter TMZ efficacy in sensitive cells. In a co-culture assay, GFP-labeled parental cells became insensitive to TMZ when cocultured with mCherry-labeled TMZ-resistant cells (1:1 ratio). The results indicated that factors secreted by TMZ-resistant GBM cells promoted the resistance phenotype in sensitive GBM cells (Fig. 1G). To explore whether exosomes play a critical role in this effect, we reduced exosome production through interference with Rab27a/b expression by RNA interference (RNAi) or pharmacological inhibition of neutral sphingomyelinase-2 (nSMase) with GW4869. Interestingly, CM from LN229/TMZ-R3rd cells with Rab27a/b knockdown or GW4869 treatment failed to confer TMZ resistance on recipient cells, indicating the critical role of exosomes in the transmission of resistance (Additional file 1: Fig. S1C). Cell-secreted exosomes and capped cargoes can be internalized by neighboring cells. In a coculture system, CD63-GFP-labeled TMZ-resistant cells delivered exosomes to the adjacent mCherry-labeled TMZ-sensitive cells (Fig. 1H). Next, we tested the effect of resistant cell-secreted exosomes on the therapeutic sensitivity of GBM cells and found that exosomes released from resistant GBM cells (Res-exo), but not those released from sensitive GBM cells (Sen-exo), enhanced the resistance of recipient GBM cells to TMZ in vitro (Fig. 1I and Additional file 1: Fig. S1D).

Patient-derived organoids (PDOs) can be used to predict responses to chemotherapy. Here, we successfully established PDOs and used them to investigate drug responses after treatment. PDOs were not sensitive to TMZ when pre-incubated with Res-exo (Fig. 1J). We administered mice with exosomes derived from TMZ-resistant and parental cells during chemotherapy. As shown in Fig. 1K, exosomes derived from TMZ-resistant cells but not parental cells significantly inhibited the response of GBM xenografts to TMZ, as determined by comparing the tumor sizes and mouse survival times. In addition, exosome-conferred resistance was sustained for at least 7 days in recipient cells after the removal of Res-exo and could not be reversed by Sen-exo (Additional file 1: Fig. S1E, F). These data suggest that the resistance of recipient cells conferred by Res-exo is sustainable.

### Intercellular transmission of circCABIN1 by exosomes significantly dampens the response of sensitive cells to TMZ

**Fig. 2:**
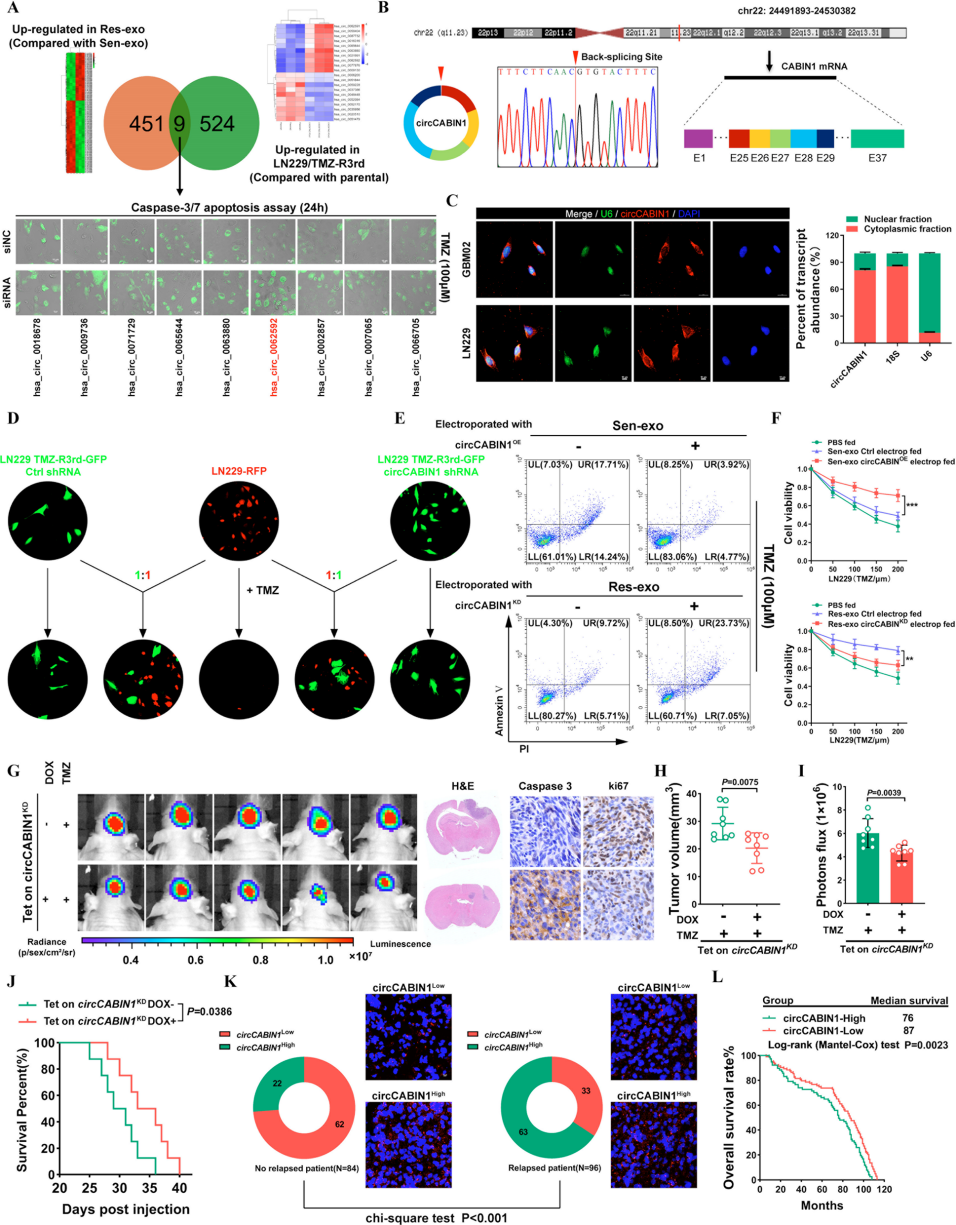
Intercellular transfer of circCABIN1 by exosomes disseminates temozolomide resistance. **A** Upper, Screening differentially expressed circRNA in TMZ-R cells and Res-exo by circRNA deep-sequencing. Lower, LN229 cells were transfected with siNC or siRNA and treated with TMZ (100 μM), and apoptosis was detected by caspase3/7 assay. Scale bar, 10 μm. **B** Explanation of the illustrated genomic loci of CABIN1, and the verification strategy for the circular exon 25–29 (circCABIN1). Sanger sequencing following PCR was used to show the “head-to-tail” splicing of circCABIN1 **C** Left, localization of circCABIN1 (red) in cells using fluorescence in situ hybridization (FISH). U6 probe coupled with Alexa Fluor™ 488 (green) and nuclei were counterstained with DAPI (blue). Scale bar, 10 μm. Right, nuclear and cytoplasmic fractions were isolated. circCABIN1 was mainly localized in cytoplasm. **D** TMZ-R3rd-GFP cells transfected with Ctrl shRNA or circCABIN1 shRNA co-cultured with parental cells (mCherry) at a ratio of 1:1 with TMZ treatment (100uM) for 4 days. Scale bar, 10 μm. **E** LN229 cells administrated with Sen-exo/Res-exo electroporated with circCABIN1^OE/KD^ and TMZ were subjected to FACS to detect apoptosis. **F** The effect of Sen-exo/Res-exo electroporated with circCABIN1^OE/KD^ on LN229 cells by CCK-8 assay. **G-J** Nude mice were orthotopically xenografted with Tet on circCABIN1 KD GBM cells (2 × 10^6^ cells) and treated with DOX-diet and intraperitoneally with TMZ (40 mg/kg) daily. Left, IVIS detects bioluminescence signals. Right, representative images of H&E and cleaved Caspase-3 and ki67 IHC in GBM sections of indicated groups. Quantification of tumor size (**H**), and quantification of bioluminescent imaging signal intensities (**I**), and survival rate (**J**) of mice in indicated groups. **K** Expression of circCABIN1 in relapsed or non-relapsed patients and representative immunofluorescence images in GBM section of indicated groups. **L** Kaplan–Meier analysis of OS in the high and low circCABIN1 groups according to the median circCABIN1 level in pre-therapy plasma (p = 0.0023). Results are presented as mean ± SD. **p < 0.01, and ***p < 0.001

To identify the critical circRNAs that contribute to the dissemination of drug resistance characteristics, circRNA deep sequencing was performed in LN229/TMZ-R3rd and parental cells. We also compared the circRNA transcriptome profiles of exosomes isolated from LN229/TMZ-R3rd cells (i.e., Res-exo) with those of exosomes derived from the parental LN229 cell line (i.e., Sen-exo). The circos plot and heatmap show the distribution and expression profiles of the detected and differentially expressed circRNAs on human chromosomes (Additional file 1: Fig. S2A, B). Comparative analysis revealed that 9 circRNAs were significantly upregulated in both LN229/TMZ-R3rd cells (compared with the parental cells) and Res-exo (compared with Sen-exo). The caspase-3/7 apoptosis assay revealed that the specific siRNA targeting hsa_circ_0062592 had the most significant chemosensitizing effect on GBM cells (Fig. 2A). According to circBase (http://www.circbase.org/), the circRNA hsa_circ_0062592 is derived from exons 25–29 of the CABIN1 transcript and forms a 960 nt circular transcript hereafter called circCABIN1. The back-spliced junction of circCABIN1 was amplified and confirmed by Sanger sequencing, and the sequence was consistent with that in the circBase database (Fig. 2B). We then further examined the circular characteristics of circCABIN1. Using cDNA and genomic DNA as templates, reverse transcription PCR (RT-PCR) analyses using convergent and divergent primers showed that circ CABIN1 was amplified by divergent primers in cDNA but not in genomic DNA (Additional file 1: Fig. S2C). In addition, circCABIN1 was more stable than linear CABIN1 mRNA upon RNase-R/Actinomycin D treatment (Additional file 1: Fig. S2D, F). Moreover, we utilized a Cy3-labeled circCABIN1-specific probe to target the junction region and RNA fluorescence in situ hybridization (FISH) demonstrated that circCABIN1 was localized predominately in the cytoplasm. The nuclear/cytoplasm fractionation further confirmed that circCABIN1 was expressed mainly in the cytoplasm (Fig. 2C).

We further examined whether exosome-transmitted circCABIN1 can confer a resistance phenotype on recipient GBM cells. Specifically, knockdown of circCABIN1 in resistant cells suppressed the ability of the cocultured parental cells to acquire resistance (Fig. 2D). Moreover, parental cells incubated directly with exosomes derived from TMZ-resistant cells displayed reduced sensitivity to TMZ, which could be abrogated in the recipient cells by treatment with short hairpin RNA (shRNA) against circCABIN1 (Additional file 1: Fig. S2G). GBM cells exposed to Sen-exo electroporated with circCABIN1^OE^ also exhibited a poor response to TMZ, excluding the involvement of factors other than circCABIN1 in exosomes. Res-exo electroporated with the circCABIN1^KD^ construct significantly decreased the ability of the cocultured parental cells to acquire resistance (Fig. 2E, F).

To eliminate the effect of circCABIN1 on tumorigenesis, we constructed an LN229 cell line with Tet-on inducible circCABIN1 knockdown. Knockdown of circCABIN1 induced by doxycycline (DOX) treatment significantly sensitized GBM tumor-bearing mice to TMZ, as indicated by the decreases in the bioluminescence signal intensity and tumor volume. Survival analysis showed that circCABIN1 knockdown significantly increased the median survival time of mice from 30.0 (DOX-) to 34.5 (DOX +) days. The above findings revealed that downregulation of circCABIN1 promotes TMZ sensitivity. Ki-67 and caspase-3 staining showed that circCABIN1 knockdown inhibited tumor cell proliferation and induced cell apoptosis in tumor tissues (Fig. 2G–J).

Moreover, circCABIN1 levels were increased in GBM patients who eventually experienced relapse. When we divided the patients into the high and low circCABIN1 expression groups (with the median expression value as the cutoff), the proportion of patients with high circCABIN1 expression was significantly lower in the non-relapsed patient group than in the relapsed patient group (Fig. 2K). These data suggest that circCABIN1 may be associated with recurrence in glioma patients. Kaplan‒Meier survival analysis showed that high circCABIN1 levels in glioma patients were correlated with reduced overall survival (OS) (Fig. 2L). Moreover, the level of linear CABIN1 mRNA had no effect on OS in GBM patients, according to analysis of the CGGA, TCGA, Gravendeel and Rembrandt databases (Additional file 1: Fig. S2H).

### EIF4A3 increases the biogenesis of circCABIN1 by juxtaposing the circularized exons

**Fig. 3:**
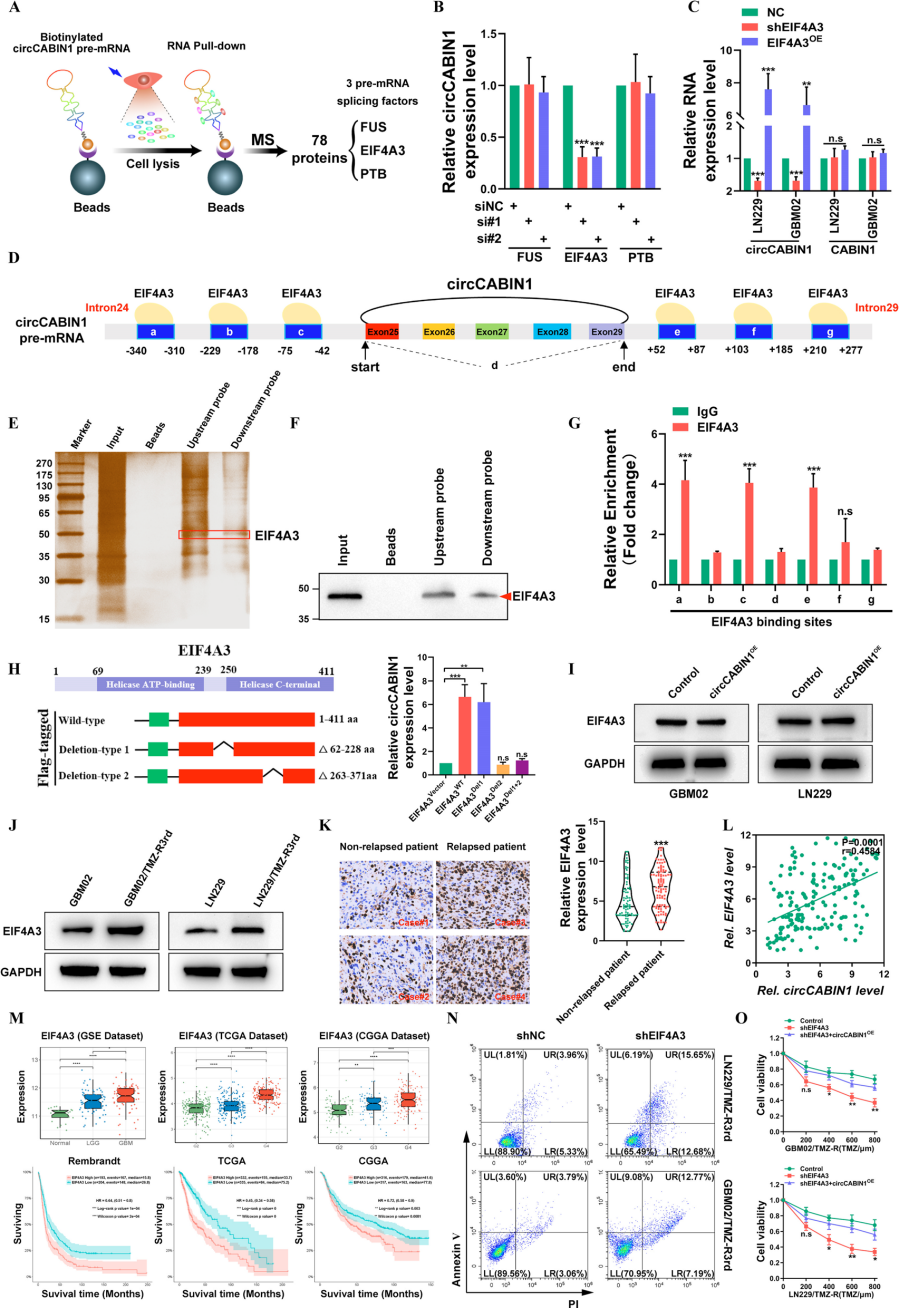
EIF4A3 promotes the biogenesis of circCABIN1. **A** Schematic image. Screening of RNA binding proteins by mass spectrometry. **B** The expression of circCABIN1 in LN229 cells was analyzed by qRT-PCR after indicated transfection. **C** The expression of circCABIN1 was analyzed by qRT-PCR after overexpress or knockdown EIF4A3. **D** Schematic image. Binding site of EIF4A3 and circCABIN1 pre-mRNA. **E–F** Pull-down silver staining and western blotting were used to demonstrate the interaction between EIF4A3 and the circCABIN1 pre-mRNA upstream and downstream region in LN229 cells. **G** RIP assay to verify EIF4A3 binding at the putative sites. **H** Schematic image. Construction of wild-type and deletion mutant expression plasmids for EIF4A3, and the expression of circCABIN1 in LN229 cells was analyzed by qRT-PCR after indicated transfection. **I** The expression of EIF4A3 was analyzed by western blot after overexpressed circCABIN1. **J** The expression of EIF4A3 was analyzed by western blot in TMZ resistant and parental cells. **K** The expression of EIF4A3 in relapsed or non-relapsed patients and representative EIF4A3 IHC images in GBM section of indicated groups. **L** Pearson correlation analysis of circCABIN1 and EIF4A3 (p = 0.0001). **M** The expression of EIF4A3 in different stages GBM and Kaplan–Meier analysis of OS in the high and low EIF4A3 groups in public database. **N** TMZ resistant cells administrated with shNC or shEIF4A3 were subjected to FACS to detect apoptosis. **O** The effect of shEIF4A3 and shEIF4A3 plus circCABIN1^OE^ on TMZ resistant cells by CCK-8 assay. Results are presented as mean ± SD. *p < 0.05, **p < 0.01, ***p < 0.001 and ****p < 0.0001. NS, not significant

To identify the potential factor(s) involved in circCABIN1 cyclization, an RNA pull-down assay was performed using circCABIN1 pre-mRNA, which was prepared via in vitro transcription, and the products were then used for mass spectrometry-based proteomic analysis. A total of 78 proteins, including 3 pre-mRNA splicing factors (PTB, EIF4A3 and FUS), were identified as potent circCABIN1 pre-mRNA-interacting proteins (Fig. 3A). Further qRT‒PCR analysis revealed that silencing EIF4A3 significantly reduced but overexpressing EIF4A3 increased circCABIN1 expression in GBM cells. Moreover, overexpressing EIF4A3 did not affect the expression level of CABIN1, suggesting that EIF4A3 might be involved in circCABIN1 cyclization (Fig. 3B, C).

We then examined whether EIF4A3 directly interacts with circCABIN1 pre-mRNA. CircInteractome (https://circinteractome.nia.nih.gov/index.html) analysis revealed 6 putative EIF4A3 binding sites in the upstream and downstream regions of circCABIN1 pre-mRNA (Fig. 3D). Then, we performed an RNA pulldown assay using specific biotinylated probes. As shown in Fig. 3E and F, EIF4A3 bound to both the upstream and downstream flanking sequences of circCABIN1. In addition, the RIP assay showed that endogenous EIF4A3 bound to two upstream putative binding sites (named a and c) and one downstream putative binding site (named e) but not the back-spliced junction site (named d) (Fig. 3G). Moreover, we found that circCABIN1 has binding sites located in introns 24 and 29 of CABIN1 pre-mRNA. EIF4A3 can be inferred to promote circCABIN1 biogenesis by bringing exons 25 and 29 into close proximity. Therefore, these results demonstrate that EIF4A3 directly binds to circCABIN1 pre-mRNA and induces circCABIN1 cyclization.

To further elucidate the binding sites of EIF4A3 in the circCABIN1 pre-mRNA transcript, we performed a screen with the catRAPID algorithm and identified peptides containing aa 62–228 and aa 263–371 of EIF4A3 as two possible interaction regions. Consequently, we constructed wild-type EIF4A3 and EIF4A3 deletion mutant (Δ62-228 aa and Δ263-371 aa) expression plasmids. Ectopic expression of wild-type EIF4A3 or deletion-type 1 (Δ62-228 aa) but not deletion-type 2 (Δ263-371 aa) induced the expression of circCABIN1 in GBM cells (Fig. 3H). In contrast, silencing circCABIN1 did not change the EIF4A3 expression level (Fig. 3I). These results suggest that this domain (aa 263–371) is indispensable for EIF4A3-mediated regulation of circCABIN1 cyclization.

As expected, EIF4A3 was upregulated in TMZ-resistant cell lines (Fig. 3J). In addition, elevated EIF4A3 expression was identified in samples of relapsed glioma compared to samples of non-relapsed glioma, and a positive correlation between circCABIN1 and EIF4A3 expression was identified in glioma samples (Fig. 3K–L). Moreover, analysis of the CGGA, TCGA and the GSE4290 dataset indicated that EIF4A3 expression was moderately increased in lower-grade glioma (LGG) tissues but strongly elevated in GBM tissues. Importantly, patients with high EIF4A3 expression had a shorter OS time than those with low EIF4A3 expression in the CGGA, TCGA and Rembrandt databases (Fig. 3M). An apoptosis assay showed that silencing EIF4A3 increased the cytotoxicity of TMZ in LN229/TMZ-R3rd and GBM02/TMZ-R3rd cells, whereas ectopic expression of circCABIN1 promoted the TMZ resistance phenotype (Fig. 3N, O). Therefore, these results demonstrate that EIF4A3 directly binds to circCABNIN1 pre-mRNA and induces circCABNIN1 cyclization.

### Control of cancer stemness signature via circCABIN1

**Fig. 4:**
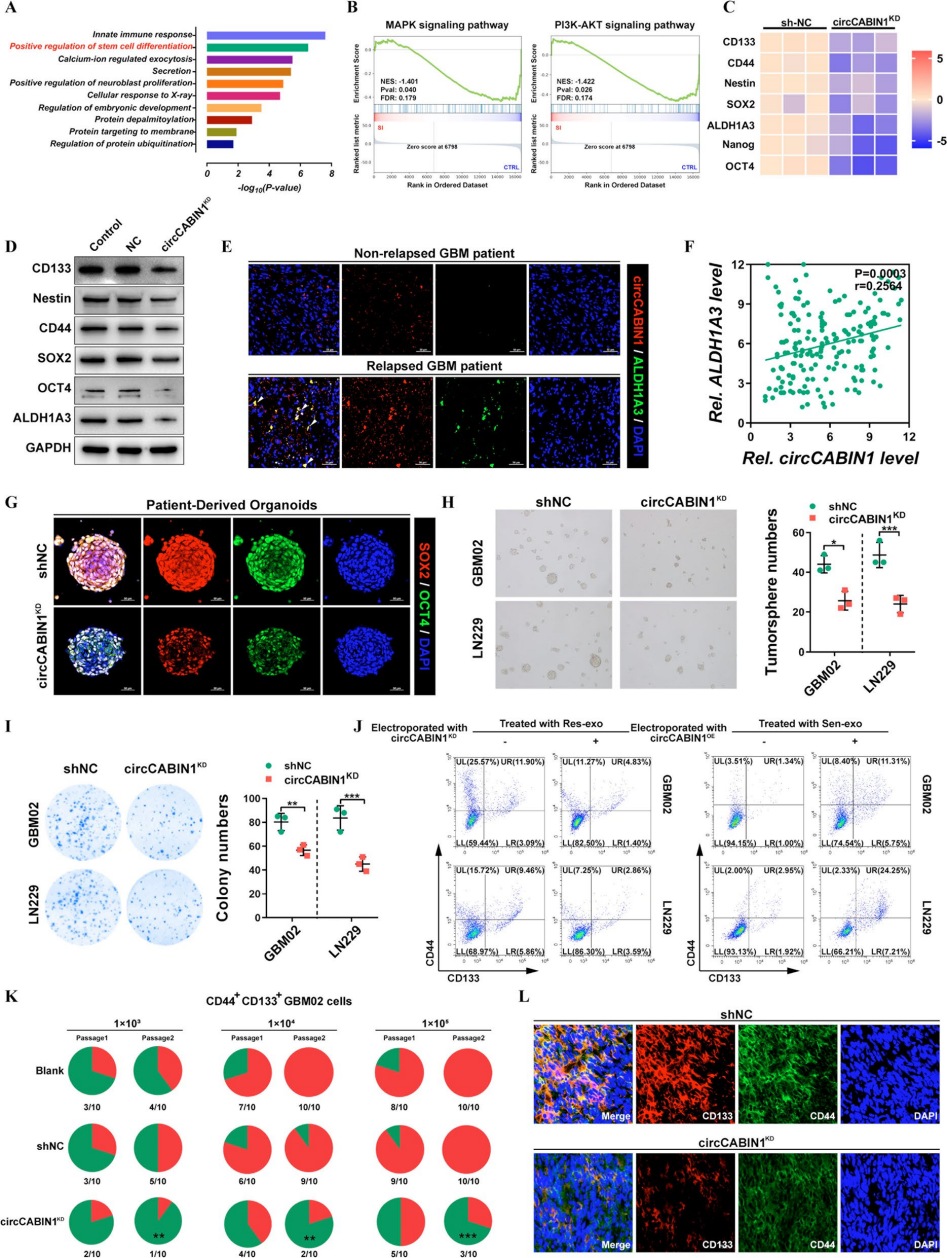
circCABIN1 promotes cancer stemness. **A** KEGG pathway analysis demonstrated that positive regulation of stem cell differentiation was involved and might be the downstream of circCABIN1. **B** GSEA analysis showed that differential genes were mainly enriched in several key stem cell related signal pathways, such as PI3K-Akt and MAPK pathway. **C** Heat map was performed to analyze the changed key stemness molecules after knockdown of circCABIN1. **D** The expression of key stemness molecules in LN229 cells were analyzed by western blot after knockdown of circCABIN1. **E** Fluorescence images showed the expression of circCABIN1 (Red) and ALDH1A3 (Green) in patient samples. Nuclei were counterstained with DAPI (Blue). Scale bar, 50 μm. **F** Pearson correlation analysis of circCABIN1 and ALDH1A3 (p = 0.0003). **G** Fluorescence images of patient-derived organoids was to confirm the expression of SOX2 (red) and OCT4 (green) after knockdown of circCABIN1. Nuclei were counterstained with DAPI (blue). Scale bar, 50 μm. **H-I** Tumorsphere formation assay and colony formation detected GSC’s self-renewal and proliferation ability after knockdown of circCABIN1. **J** GBM cells administrated with Sen-exo or Res-exo electroporated with circCABIN1^OE/KD^ were subjected to FACS to detect the proportion of CD44^+^CD133^+^ cells. **K** Incidences of tumorigenesis of CD45^+^CD133^+^ primary GBM cells (GBM02) infected with circCABIN1^KD^ in serial transplantation models. **P < 0.01 compared with untreated primary GBM cells in the first inoculation by Fisher’s exact test. **L** Fluorescence images showed the expression of CD133 (red) and CD44 (green) of brain frozen section from orthotopic GBM xenograft mice in indicated groups. Nuclei were counterstained with DAPI (blue). Scale bar, 10 μm. Results are presented as mean ± SD. *p < 0.05, **p < 0.01 and ***p < 0.001

To explore the underlying mechanisms by which circCABIN1 KD promotes TMZ sensitivity, we performed whole-transcriptome analysis of LN229 cells upon circCABIN1 KD using RNA sequencing. Kyoto Encyclopedia of Genes and Genomes (KEGG) pathway enrichment analysis revealed that circCABIN1 KD affected the expression of genes enriched in the “pluripotency of stem cells” pathway (Fig. 4A). Furthermore, Gene Set Enrichment Analysis (GSEA) revealed that these differentially expressed genes were enriched mostly in several key stemness-related signaling pathways, such as the PI3K-AKT and MAPK pathways, which regulate dedifferentiation (Fig. 4B). The expression profiles of CD133, CD44, Nestin, SOX2, Nanog, ALDH1A3 and OCT4, which are associated with glioma stem cell (GSC) signatures, were visualized in a heatmap (Fig. 4C). Western blot analysis confirmed that circCABIN1 KD reduced the expression of CD133, Nestin, CD44, SOX2, OCT4 and ALDH1A3 in GBM cells (Fig. 4D). Similarly, we found that circCABIN1 preferentially expressed in GSCs (ALDH1A3^+^) in relapsed patient tissues (Fig. 4E). Additionally, Pearson correlation analysis revealed that the expression of circCABIN1 was positively correlated with the expression of ALDH1A3 in 180 glioma patient tissues (Fig. 4F). Moreover, circCABIN1 expression was upregulated in sorted CD44^+^CD133^+^ primary GBM cells and in spheres formed from primary GBM cells (Additional file 1: Fig. S3A, B). Organoids form miniature replicas of the host organs by allowing the differentiation and self-organization of stem cells to acquire organ-specific cellular functions and structures. To evaluate circCABIN1 KD-mediated suppression of cancer stemness, PDOs were allowed to differentiate from stem cells in the presence or absence of a circCABIN1 shRNA adenovirus. circCABIN1 KD treatment suppressed the expression of both SOX2 and OCT4, showing that circCABIN1 KD eliminates GSCs (Fig. 4G). The tumorsphere formation assay is a simple method to evaluate the self-renewal of GSCs in vitro. circCABIN1 KD also significantly inhibited the tumorsphere-forming ability of CD44^+^CD133^+^ GBM cells (Fig. 4H). Moreover, the in vitro limiting dilution assay showed that circCABIN1 KD inhibited the sphere-forming ability of CD44^+^CD133^+^ GSCs (Additional file 1: Fig. S3C). The colony formation assay showed that circCABIN1 KD reduced colony numbers compared with those in the negative control shRNA (shNC) group (Fig. 4I). Moreover, GBM cells incubated directly with exosomes derived from TMZ-resistant cells displayed enhanced expression of stem cell markers, which could be abrogated by exposure to Res-exo electroporated with siRNA against circCABIN1. GBM cells exposed to Sen-exo electroporated with the circCABIN1^OE^ construct also increased the proportion of CD44^+^CD133^+^ cells (Fig. 4J). In addition, exosomes derived from parental cells did not affect the characteristics of GSCs (Additional file 1: Fig. S3D).

Furthermore, serial transplantation was performed by subcutaneous injection of CD44^+^CD133^+^ cells isolated from primary GBM cells into a second and a subsequent third batch of mice. circCABIN1 KD substantially suppressed tumorigenicity upon in vivo serial passaging of sorted CD44^+^CD133^+^ cells, suggesting that circCABIN1 KD impaired the tumor formation ability of GSCs (Fig. 4K). Consistent with this finding, the number of CD44^+^CD133^+^ tumor cells was significantly decreased in circCABIN1^KD^ mice compared with shNC mice (Fig. 4L). Collectively, these results indicate that circCABIN1 KD impairs the self-renewal and maintenance of GSCs and suppresses tumor propagation.

### CircCABIN1 directly binds to miR-637 and acts as a competing endogenous RNA

**Fig. 5:**
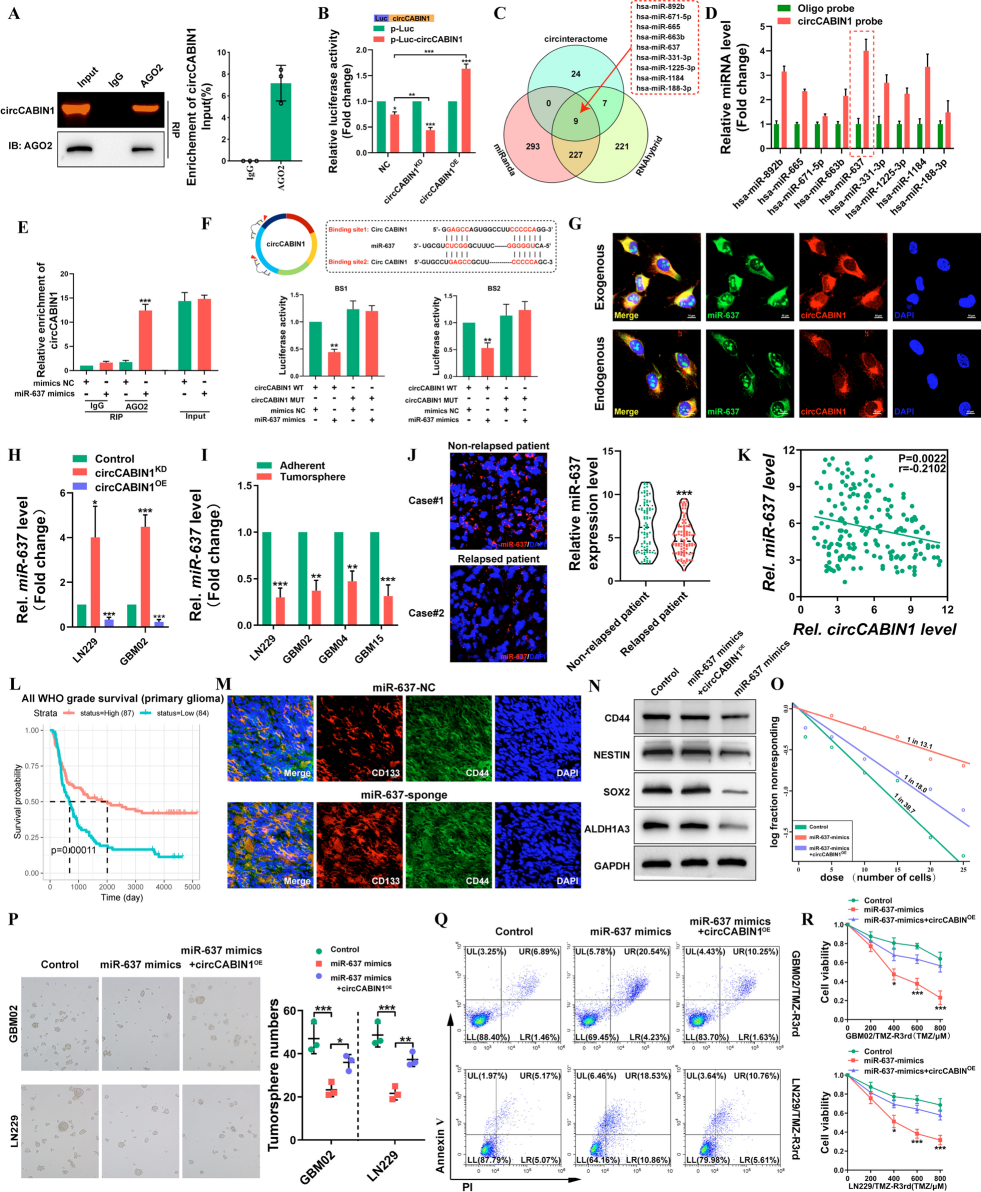
circCABIN1 acted as a sponge of miR-637 in GBM. **A** RIP assay verified AGO2 binding with circCABIN1. **B** A luciferase reporter assay was conducted to measure the luciferase activity of p-luc circASAP1 in HEK-293 T cells cotransfected with the circCABIN1^KD^ or circCABIN1^OE^. **C** The venn diagram showed that 9 miRNAs were changed together in three groups. **D** Pull down and qRT-PCR assay verified 9 miRNA binding with circCABIN1 probe. **E** RIP assay verified miR-637 binding with AGO2. **F** Schematic diagram of the circCABIN1 and miR-637 binding sites and luciferase reporter assay verified circCABIN1 binding with miR-637 mimics. **G** The co-localization of circCABIN1 (red) and exogenous / endogenous miR-637 (green) in LN229 cells using fluorescence in situ hybridization (FISH). Nuclei were counterstained with DAPI (blue). Scale bar, 10 μm. **H** The expression of miR-637 in GBM cells were analyzed by qRT-PCR after knockdown or overexpression of circCABIN1. **I** The expression of miR-637 in TMZ-resistant and adherent cells by qRT-PCR. **J** The expression of miR-637 in relapsed or non-relapsed patients by fluorescence confocal and qRT-PCR. **K** Pearson correlation analysis of circCABIN1 and miR-637 (p = 0.0022). **L** Kaplan–Meier analysis of OS in the high and low miR-637 groups in CGGA (p = 0.00011). **M** Fluorescence images showed the expression of CD133 (red) and CD44 (green) of brain frozen section from orthotopic GBM xenograft mice in indicated groups. Nuclei were counterstained with DAPI (blue). Scale bar, 10 μm. **N-P** Western blot, limiting dilution analysis and tumorsphere formation assays detected the stem cell properties of cells transfected with control vector, miR-637 mimics alone or miR-637 mimics plus circCABIN1^OE^. **Q-R** GBM cells administrated with control vector, miR-637 mimics alone or miR-637 mimics plus circCABIN1^OE^ were subjected to FACS and CCK-8 assays. Results are presented as mean ± SD. *p < 0.05, **p < 0.01 and ***p < 0.001

Previous studies have shown that circRNAs can function as miRNA sponges, bind to RNA-binding proteins (RBPs), act as transcription factors, or be translated into proteins [23, 24]. Because circCABIN1 was shown to be localized in the cytoplasm, we first investigated whether circCABIN1 can act as a ceRNA for miRNAs. The RIP assay confirmed that circCABIN1 can directly interact with Argonaute-2 (Ago2), a core component of the RNA-induced silencing complex (Fig. 5A). A luciferase reporter gene with a circCABIN1 fragment was constructed and inserted. Subsequently, knockdown and overexpression of endogenous circCABIN1 expression further decreased and increased luciferase activity, respectively (Fig. 5B). These results suggest that circCABIN1 might serve as a binding platform for Ago2 and miRNAs.

To investigate novel downstream molecules, three bioinformatics databases, miRanda, RNAhybrid and CircInteractome were used as tools to predict the candidate target miRNAs possibly binding to the circCABIN1 sequence (Fig. 5C). For a circRNA pulldown assay, a dedicated biotinylated circCABIN1 probe was used, and circCABIN1-related RNAs were purified prior to qRT–PCR. Compared with control group samples, samples pulled down with the circCABIN1-specific probe showed notable enrichment of miR-637 (Fig. 5D). Subsequently, an anti-AGO2 RIP assay was performed. Compared with IgG, the anti-AGO2 antibody efficiently pulled down miR-637 and circCABIN1; furthermore, the amount of immunoprecipitated circCABIN1 was significantly higher in the miR-637-overexpressing samples than in the NC samples (Fig. 5E). Additionally, bioinformatic analysis showed that circCABIN1 contains two sequences complementary to the miR-637 seed regions. The luciferase reporter assay showed that only the miR-637 mimic markedly suppressed psiCHECK2-circCABIN1 luciferase activity, whereas luciferase activity was not significantly changed when the miR-637 binding sites were mutated (Fig. 5F). Moreover, FISH indicated that miR-637 colocalized with both endogenous and exogenous circCABIN1 in GBM cells (Fig. 5G). In general, these results demonstrate that circCABIN1 physically interacts with miR-637 in GBM cells. Knockdown or overexpression of circCABIN1 resulted in up- or downregulation of miR-637, respectively, in GBM cells (Fig. 5H). Notably, miR-637 expression was more substantially decreased in TMZ-resistant GBM cells than in the corresponding parental cells, in tumorspheres than in non-spheroid cells, and in GSCs than in non-GSCs (Fig. 5Iand Additional file 1: Fig. S4A, B). Subsequently, we evaluated the expression level of miR-637 in glioma tissues. miR-637 was downregulated in relapsed glioma tissues compared with non-relapsed glioma tissues (Fig. 5J). In addition, a negative correlation between circCABIN1 and miR-637 expression was identified (r = -− 0.2102, P = 0.0022, Fig. 5K). Moreover, in the CGGA database, patients with higher expression levels of miR-637 tended to have longer survival times (Fig. 5L). Sponge lentivirus for miR-637 knockdown increased the proportion of CD44^+^CD133^+^ GSCs in GBM tumor-bearing mice (Fig. 5M).

To further investigate whether the binding of miR-637 and circCABIN1 regulates the functions of cancer cells, we co-transfected GBM02 and LN229 cells with the circCABIN1 overexpression vector and miR-637 mimic. The results of the limiting dilution, colony formation and tumorsphere formation assays revealed that the miR-637 mimic significantly reversed the enhancement of stem cell properties induced by circCABIN1 overexpression (Fig. 5N–P and Additional file 1: Fig. S4C). Moreover, the miR-637 mimic accelerated TMZ-induced apoptosis, while combined circCABIN^OE^ partially inhibited apoptosis in LN229/TMZ-R3rd and GBM02/TMZ-R3rd cells (Fig. 5Q, R). Taken together, these findings indicate that circCABIN1 knockdown reduces the malignancy of GBM cells by targeting miR-637.

### Olfactomedin-like 3 (OLFML3) is a downstream target of the circCABIN1/miR-637 signaling axis

**Fig. 6:**
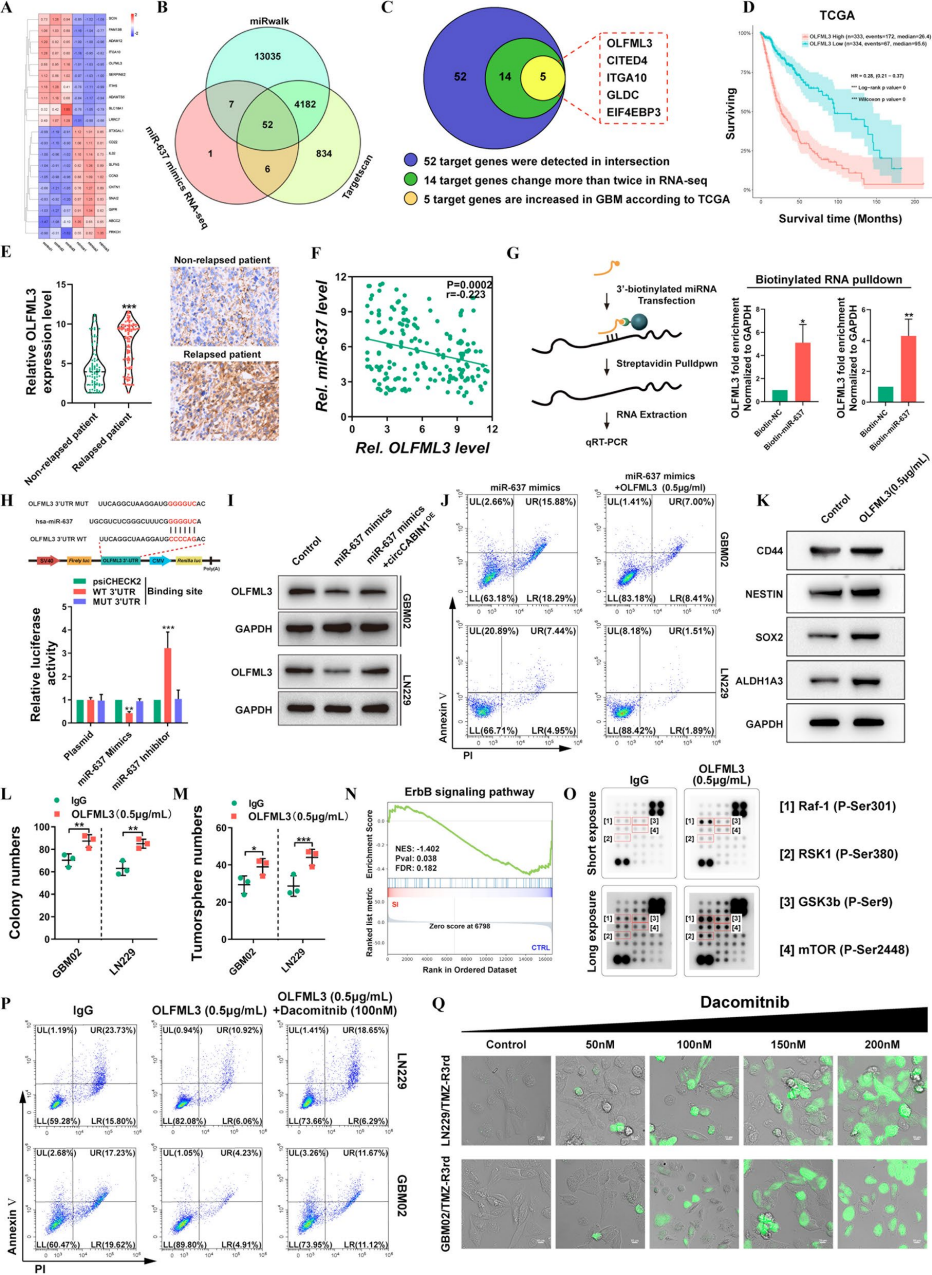
OLFML3 was a downstream target of the circCABIN1/miR-637 signalling axis. **A-C** 5 possible target genes were screened through RNA-seq, miRwalk, Targetscan and TCGA database. Heat map was performed to analyze the changed genes after transfected with miR-637 mimics (**A**). The venn diagram showed that 52 genes were changed together in three groups (**B**). Schematic illustration of genes significantly increased in GBM (**C**). **D** Kaplan–Meier analysis of OS in the high and low OLFML3 groups in TCGA. (p < 0.001). **E** The expression of OLFML3 in relapsed or non-relapsed patient and representative images of IHC in GBM sections of indicated groups. **F** Pearson correlation analysis of OLFML3 and miR-637 (p = 0.0002). **G** Pull down and qRT-PCR assay verified miR-637 binding with OLFML3 mRNA. **H** Schematic diagram of the OLFML3 mRNA and miR-637 binding sites and luciferase reporter assay verified OLFML3 mRNA binding with miR-637. **I** The expression of OLFML3 after transfected with miR-637 mimics alone or miR-637 mimics plus circCABIN1^OE^ were analyzed by western blot in LN229 cells. **J** GBM cells administrated with miR-637 mimics alone or miR-637 mimics plus OLFML3 recombinant protein (0.5 μg/mL) were subjected to FACS to detected apoptosis. **K-M** Western blot, colony formation and tumorsphere formation assays detected the stem cell properties after treated with OLFML3 recombinant protein (0.5 μg/mL). **N** GSEA analysis showed that differential genes were mainly enriched in ErbB signal pathway (p = 0.038). **O** Protein array assay detected ErbB-related signalings after OLFML3 recombinant protein treatment (0.5 μg/mL). **P** GBM cells administrated with OLFML3 recombinant protein (0.5 μg/mL) alone or OLFML3 recombinant protein plus Dacomitnib (100 nM) were subjected to FACS to detected apoptosis. **Q** TMZ-resistance cells treated with dacomitnib with concentration gradient, and apoptosis was detected by caspase3/7 experiment. Scale bar, 10 μm. Results are presented as mean ± SD. *p < 0.05, **p < 0.01 and ***p < 0.001

To determine the downstream target genes of the circCABIN1/miR-637 signaling axis, RNA-seq combined with bioinformatic analysis using TargetScan and miRWalk was performed to analyze the downstream targets of miR-637 (Fig. 6A, B). The data revealed 52 possible genes targeted by miR-637. After filtering out differentially expressed mRNAs with an expression fold change of less than two in the RNA-seq data and genes whose expression was not elevated in GBM tumor tissues compared with the corresponding normal tissues in the TCGA database, EIF4EP3, GLDC, ITGA10, CITED4 and OLFML3 were selected for further analysis (Fig. 6C). Survival analysis in TGCA showed that a high level of only OLFML3 was correlated with poor prognosis in GBM patients (Fig. 6D and Additional file 1: Fig. S5A). Similar results were observed in the CGGA, Gravendeel and Rembrandt databases (Additional file 1: Fig. S5B). The upregulation of OLFML3 in GBM was verified in the GSE4290, GSE50161, GSE59612 and GSE11260 datasets (Additional file 1: Fig. S5C). Moreover, OLFML3 was highly expressed in glioma patients with relapse compared with glioma patients without relapse (Fig. 6E). Correlation analysis showed that the expression level of OLFML3 in glioma tissues was negatively correlated with that of miR-637 (Fig. 6F).

To obtain more direct evidence for the interaction of miR-637 with OLFML3 mRNA, we performed a miRNA pulldown assay. OLFML3 mRNA was significantly enriched in the miR-637 mimics precipitate relative to the miR-NC precipitate (Fig. 6G). The miR-637 mimic significantly decreased and the miR-637 inhibitor significantly increased the luciferase activity in cells expressing the WT plasmid, but these effects were not observed in cells expressing the mutant plasmid (Fig. 6H). To further clarify the regulatory role of circCABIN1 in the miR-637/OLFML3 axis, we overexpressed miR-637 in GBM02 and LN229 cells in the presence or absence of circCABIN1^OE^. miR-637 overexpression diminished OLFML3 expression, whereas transfection with the circCABIN1^OE^ construct abolished this effect (Fig. 6I). Further studies confirmed that the recombinant OLFML3 protein neutralized the ability of the miR-637 mimic to enhance TMZ sensitivity (Fig. 6J). To clarify the mechanism of OLFML3-mediated TMZ resistance, we performed RNA-seq to compare the gene profiles of LN229 cells with high and low OLFML3 expression. Gene Ontology (GO) enrichment analysis showed that the differentially expressed mRNAs in OLFML3^High^ compared with OLFML3^Low^ LN229 cells were enriched in gene sets related to cancer stem cell (CSC) maintenance, consistent with the promoting role of circCABIN1 in GSCs (Additional file 1: Fig. S5D). We searched the CGGA database to check the correlations between the expression of OLFML3 and that of these key stemness molecules, and the results revealed positive correlations between OLFML3 and CD44, SOX2, Nestin, ALDH1A3 (Additional file 1: Fig. S5E). Subsequently, the expression levels of GSC marker genes, including CD44, SOX2, Nestin and ALDH1A3, were examined and found to be markedly increased after treatment with recombinant OLFML3 protein (Fig. 6K). Furthermore, overexpression of OLFML3 promoted colony formation (Fig. 6L, M and Additional file 1: Fig. S5F). However, the mechanism of OLFML3 in GSC maintenance remains unclear.

GSEA indicated that the differentially expressed genes induced by OLFML3 knockdown were enriched in the ErbB signaling pathway (Fig. 6N). Given the crucial role of the ErbB signaling pathway in the development of GBM, we investigated whether OLFML3 can modulate the ErbB signaling pathway. ErbB-related signaling was analyzed using a protein array after treatment with recombinant OLFML3 protein. The array contained 18 site-specific and phospho-specific antibodies. In the array, the signals, including those for phospho-Raf-1 (Ser301), phospho-RSK1 (Ser380), phospho-GSK-3β (Ser9) and phospho-mTOR (Ser2448), were markedly activated by treatment with OLFML3 (Fig. 6O). Previous studies confirmed that the activation of these pathways is related to self-renewal maintenance of GSCs and drug resistance [25–27]. To explore whether the ErbB pathway mediates the function of OLFML3 in TMZ resistance, we used dacomitinib, an irreversible pan-ErbB inhibitor. Pharmacological inhibition of ErbB with dacomitinib blocked the effect of OLFML3 on TMZ resistance (Fig. 6P). Unsurprisingly, OLFML3 was highly expressed in LN229/TMZ-R3rd and GBM02/TMZ-R3rd cells (Additional file 1: Fig. S5G). Dacomitinib promoted apoptosis in TMZ-resistant cells in a dose-dependent manner (Fig. 6Q). These results indicated that OLFML3 promote TMZ resistance in an ErbB pathway-dependent manner.

### Targeted delivery of chemically modified multi-siRNAs by engineered ANG-exo sensitizes GBM cells to TMZ

Our findings indicate that EIF4A3-induced circCABIN1 functions in an important underlying mechanism in TMZ resistance through modulation of the miR-637/OLFML3 signaling pathway, which could provide pivotal potential therapeutic targets for GBM. Considering these findings, we sought to simultaneously interfere with the expression and function of these two genes (circCABIN1 and OLFML3). Then, we designed a multi-siRNA containing the sequences corresponding to si-circCABIN1 and si-OLFML3; the sequence “AUUGCAC” was used as a linker to connect si-circCABIN1 and si-OLFML3. The multi-siRNA was modified with cholesterol to increase stability and coupled to cy3 groups for tracing. Results showed that this multi-siRNA could simultaneously suppress circCABIN1 and OLFML3 expression (Additional file 1: Fig. S6A, B).

**Fig. 7:**
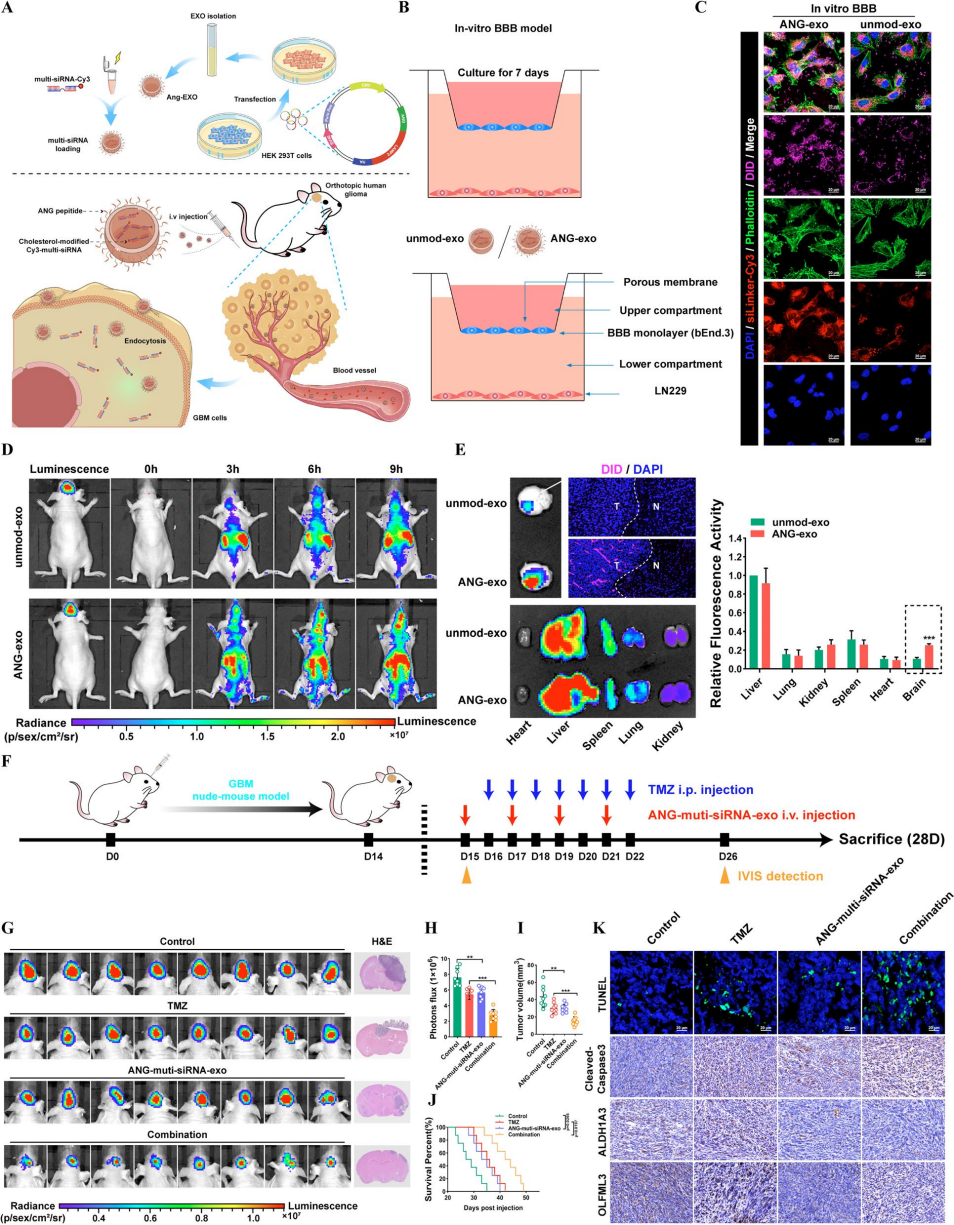
Targeted delivery of chemically modifed multi-siRNAs by engineered ANG-exo sensitized GBM cells to temozolomide. **A** Schematic image. Process of ANG-exo construction, isolation, multi-siRNA loading, animal tail vein injection. **B** Schematic image of the BBB model in-vitro. **C** Immunofluorescence images detected unmod-exo/ANG-exo uptake into LN229 cells after passing through a bEnd.3 monolayer. Scale bar, 10 μm. **D** In vivo florescence imaging of Orthotopic GBM xenograft mice at 0 h, 3 h, 6 h and 9 h time point after intravenous administration of unmod-exo / ANG-exo. **E** Left, ex vivo fluorescence images of Brain, Liver, Spleen, Lung, Heart, Kidney and frozen section of brain from mice sacrificed at 9 h post-injection. Right, fluorescence quantitative analysis of ex vivo organs of tumor-bearing mice after intravenous injection. **F** Schematic image. Time line of nude mice receiving combination therapy. **G-J** Verify the effect of combined therapy in vivo. IVIS detects bioluminescence signals. Quantification of bioluminescent imaging signal intensities (**H**), quantification of tumor size (**I**), and survival rate (**J**) of mice in indicated groups. **K** Representative images of TUNEL assay and cleaved Caspase-3, ALDH1A3 and OLFML3 IHC in GBM sections of indicated groups. Scale bar, 20 μm. Results are presented as mean ± SD. **p < 0.01 and ***p < 0.001

Chemical modification effectively improves the stability of siRNAs in blood, but the lack of targeting limits its application. Targeted delivery of siRNAs loaded in engineered exosomes was thus our first choice. Exosomes have become the focus of drug delivery research and are characterized by high biosafety, ease of preparation, and weak immunogenicity. Notably, angiopep-2 (ANG) is a promising ligand that binds specifically to the lipoprotein receptor-related protein 1 (LRP1) receptor and can improve the BBB transport efficiency of exosomes for drug delivery into the brain [28, 29]. Therefore, we designed a targeted delivery scheme based on engineered ANG-exo and the chemically modified multi-siRNA. We constructed an expression vector containing a fusion of ANG and LAMP2; LAMP2 carried the integrin-targeting ANG peptide to the exosomal membrane surface, allowing the exosomes to target GBM cells and cross the BBB (Fig. 7A).

In this study, multi-siRNAs were loaded into exosomes by electroporation. The sizes of exosomes were measured to be between 100 and 150 nm, and the encapsulation of the multi-siRNA did not have a significant effect on the size of the exosomes (Additional file 1: Fig. S6C). The morphology of unmodified exosomes (unmod-exo) and multi-siRNA-loaded exosomes (multi-siRNA-exo) was evaluated by TEM imaging. There were no significant differences between unmod-exo and multi-siRNA-exo (Additional file 1: Fig. S6D). Then, an in vitro BBB model was established to test the transcytosis and BBB penetration ability of these exosomes (Fig. 7B). As a result, unmod-exo showed minimal penetration, as slight fluorescence was observed in LN229 cells in the lower chamber. ANG modification enhanced the penetration of exosomes, while ANG-exo showed higher uptake by cells in the lower chamber (Fig. 7C). As shown in Additional file 1: Fig. S6E, ANG-multi-siRNA-exo significantly decreased the expression levels of circCABN1 and OLFML3. The effect of ANG-multi-siRNA-exo on TMZ sensitization was further confirmed both in TMZ-resistant and parental cells by CCK8 and apoptosis assays (Additional file 1: Fig. S6F).

To evaluate the brain-targeting delivery efficiency of ANG-exo by systemic injection, DiD-labeled exosomes were injected into mice through the tail vein. As shown in Fig. 7D, the fluorescence intensity in brain regions was significantly higher for ANG-exo than for unmod-exo at different time points (3, 6, 9 h) after injection. An effective tumor targeting effect focuses the drug on the tumor site, increases the efficacy of the drug and reduces the adverse effects on normal organs. First, we investigated whether the ANG-exo delivered the targeted drug to the tumor. Fluorescence accumulated mainly in the tumor after intravenous injection of ANG-exo. More importantly, ANG-exo were distributed mainly within the tumor boundary, suggesting their superior tumor targeting ability (Fig. 7E).

The in vivo antitumor effects of Ang-multi-siRNA-exo were evaluated in mice bearing orthotopic GBM tumors. Tumors were allowed to grow for 14 days, and were then treated with saline, TMZ, Ang-multi-siRNA-exo, or Ang-multi-siRNA-exo + TMZ. Luciferase bioluminescence signals in the tumors were then evaluated (Fig. 7F). Quantification of tumor bioluminescence revealed significantly greater inhibition of tumor growth by Ang-multi-siRNA-exo combined with TMZ than by TMZ alone. Moreover, notably, Ang-multi-siRNA-exo monotherapy had a slight treatment effect on GBM tumors. Importantly, mice administered both ANG-multi-siRNA-exo and TMZ survived significantly longer than those treated with ANG-multi-siRNA-exo or TMZ alone (Fig. 7G–J). Cleaved-Caspase3 staining and TUNEL in the orthotopic GBM mouse model confirmed significant cell death in mice receiving the combination therapy. Furthermore, immunohistochemical staining indicated that engineered ANG-exo that delivered the cholesterol-modified multi-siRNA effectively inhibited the expression of OLFML3. The expression level of ALDH1A3 in the combination treatment group was significantly lower than that in the TMZ group, suggesting that ANG-exo-mediated knockdown of circCABIN1 and OLFML3 reduces the generation and self-renewal of GSCs (Fig. 7K).

Safety has been a major concern in the use of nanocarriers for drug delivery. To eliminate the influence of immunodeficiency, we carried out similar experiments in BALB/c mice. Compared with the saline group, the Ang-multi-siRNA-exo group showed no obvious increases in the serum levels of alanine aminotransferase (ALT) and aspartate aminotransferase (AST). Moreover, the blood urea nitrogen (BUN) and creatinine (Cr) levels did not differ significantly among the groups. In addition, H&E staining of major organs showed no damage (Additional file 1: Fig. S6G–I). These results demonstrated the improved safety of ANG-exo-mediated multi-siRNA delivery for GBM therapy.

## Discussion

As it is the main alkylating antitumor agent for GBM treatment, multidrug resistance (MDR) to TMZ can develop after a long period of treatment, which leads to the poor prognosis of most patients with GBM [30, 31]. Several studies have proposed the activation of compensatory signaling pathways as a mechanism for the acquisition of TMZ resistance, but the principle remains unclear. In this study, we initially examined the effects of TMZ on exosome release from cancer cells. TMZ increased the release of exosomes from cancer cells in vitro and in tumor-bearing mice. We identified an uncharacterized circRNA, circCABIN1, that was highly expressed in TMZ-resistant GBM and functionally required for the resistance phenotype. circCABIN1 can be secreted from resistant cells via exosomes, transforming TMZ-sensitive cells into resistant cells and thereby transmitting drug resistance. circCABIN1 was abnormally expressed in tissue samples of relapsed GBM and associated with poor prognosis. In cancers, gain of stemness can have profound implications on tumor aggressiveness, drug responses and clinical outcomes [32]. Chemoresistance often involves the gradual loss of a differentiated phenotype and acquisition of progenitor or stem cell-like features [33]. We found that exosomal circCABIN1 drives stemness reprogramming in TMZ-sensitive cells and thereby confers chemoresistance.

The biogenesis of circRNA is regulated by specific cis-acting elements and trans-acting factors [34]. It has been shown that certain RNA-binding proteins promote circRNA expression [35]. Through RNA pull-down assay, we confirmed that EIF4A3 directly interacted with circCABIN1 pre-mRNA. Recent studies have shown that EIF4A3 facilitates circSEPT9 and circASAP1 expression by binding to their parental pre-mRNA transcripts [36, 37]. Via gain- and loss-of-function experiments, we found that EIF4A3 can promote the expression of circCABIN1. Moreover, we further confirmed that EIF4A3 can bind to the upstream and downstream flanking sites but not the backsplice junction site of circCABIN1 pre-mRNA. We also found that the aa 263–371 domain of EIF4A3 is indispensable for EIF4A3-mediated modulation of circCABIN1 cyclization. The secondary structure of pre-mRNAs brings forming exons of circRNA into close proximity and consequently promotes circRNA biogenesis. A previous study indicated that QKI targets the introns flanking the circRNA-forming exons of ROCK1 and NOTCH1 to promote cyclization [38, 39]. Consistent with these studies, we found that circCABIN1 has binding sites located in introns 24 and 29 of CABIN1 pre-mRNA that circCABIN1 biogenesis is enhanced via binding to recognition elements within introns in the vicinity of splice sites associated with circCABIN1 formation. Thus, EIF4A3 can be inferred to promote circCABIN1 biogenesis by bringing exons 25 and 29 into close proximity. The above data elucidate the mechanism by which circCABIN1 is upregulated by EIF4A3.

Thereafter, we elucidated the potential mechanism of circCABIN1 in cancer stemness acquisition and chemoresistance. Here, we first excluded the effect of circCABIN1 on its parental gene. Our RIP assay showed that only AGO2 can bind to circCABIN1. As AGO2 is very important for miRNA-mediated gene silencing, we suspected that circCABIN1 may function as a miRNA sponge. In this study, miR-637 was identified as the direct target miRNA of circCABIN1 by a circRNA pulldown assay. Decreased miRNA-637 is a marker of unfavorable prognosis in glioma. Here, luciferase reporter, RIP and FISH assays were conducted to verify the direct interaction of circCABIN1 and miR-637. In addition, transfecting the miR-637 mimic significantly rescued the effects of circCABIN1 overexpression on stemness reprogramming and chemoresistance. These and other related results indicate that circCABIN1 and miR-637 physically bind and interact with each other and are involved in regulating the stemness and chemoresistance of GBM cells.

The human glycoprotein OLFML3 is a matricellular protein with proangiogenic properties that belongs to the family of olfactomedin-domain-containing proteins [40]. Targeting OLFML3 in colorectal cancer suppresses tumor growth and angiogenesis and increases the efficacy of anti-PD1-based immunotherapy [41]. Lung carcinoma cell migration and invasion were affected following only 48 h of exposure to rhOLFML3, suggesting that OLFML3 may regulate key signaling pathways in lung carcinoma cells [42]. While exploration of the role of OLFML3 in GBM has just begun, depletion of OLFML3 in human glioma cells was found to reduced GAM infiltration and extend survival in a glioma xenograft mouse model [43]. Here, we revealed that OLFML3 is a direct target of miR-637. Furthermore, we demonstrated that circCABIN1 upregulates OLFML3 expression by abolishing the posttranscriptional suppression ability of miR-637. Functionally, recombinant OLFML3 protein impaired the sensitizing effect of the miR-637 mimic on GBM cell stemness and chemoresistance. Therefore, we revealed a new mechanism by which circCABIN1 functions as a ceRNA and weakens the endogenous inhibitory effects of miR-637 on OLFML3 in GBM. However, the mechanism by which OLFML3 regulates the stemness and chemoresistance of GBM cells remains elusive.

GSEA indicated that the differentially expressed genes induced by OLFML3 knockdown were enriched in the ErbB signaling pathway. The ErbB family of receptor tyrosine kinases consists of four closely related members: EGFR, HER2, HER3 and HER4 [44]. This family plays key roles in tumor growth, metastasis and therapeutic resistance through the activation of downstream pathways, such as the Ras/MAPK and PI3K/AKT pathways [26, 27]. We analyzed the ErbB downstream pathways in GBM cells via a protein array after treatment with recombinant OLFML3 protein. Signals corresponding to these pathways, including those of phospho-Raf-1 (Ser301), phospho-RSK1 (Ser380), phospho-GSK-3β (Ser9) and phospho-mTOR (Ser2448), were markedly activated. Previous studies have confirmed that the activation of these pathways is related to stemness maintenance and chemoresistance [45, 46]. Crosstalk between the Raf-MEK-ERK and PI3K-Akt-GSK3β signaling networks promotes chemoresistance, invasion/migration and stemness in oral cancer [25]. The RSK1 phosphorylation switch is associated with platinum resistance [47]. Inhibition of GSK-3β by miR-101 also sensitizes glioblastoma to TMZ [48]. The GSK-3β inhibitor 9-ING-41 potentiates the antitumor effects of conventional chemotherapeutic drugs against breast cancer cells [49]. A bitopic mTOR inhibitor represses CSC generation, anchorage independence, cell survival, and chemoresistance and efficiently inhibits tumorigenesis in mice [50]. Collectively, these findings imply that OLFML3 promotes stemness reprogramming and chemoresistance by activating the ErbB pathway. However, the mechanism of OLFML3 regulate the ErbB pathway is unclear. Most secreted glycoproteins play their roles through binding to corresponding receptors, such as Wnt1, Wnt3a, DDK-1 etc. [51]. Unfortunately, current research on this important molecule is still very limited. The specific receptor of OLFML3 has not been found, although there are many high-quality article articles about OLFML3 [40, 42]. Moreover, a previous study showed that OLFML3 through binding to BMP4 enhances the canonical SMAD1/5/8 signaling pathway [42]. This is consistent with general properties of the olfactomedin protein family, which are known to interact with multiple protein binding partners and regulate several cell signaling pathways. Further investigations on the possible mechanism of OLFML3 biological functions and modulation will help us develop better therapeutic strategies. Dacomitinib (Pfizer) is an irreversible second-generation pan-ErbB inhibitor. Dacomitinib showed promising clinical activity in advanced non-small cell lung cancer patients who progressed on platinum therapy and were previously treated with erlotinib [52]. Systemic administration of dacomitinib efficiently impaired EGFR signaling in vivo and affected the growth and survival of tumors formed from EGFR-amplified GBM cells, independent of the presence of different mutant receptor isoforms [53]. Moreover, dacomitinib decreased the levels of stem cell markers in the treated tumors [54]. Consistent with these results we found that pharmacological inhibition of ErbB with dacomitinib blocked the effect of OLFML3 on TMZ resistance. Surprisingly, dacomitinib promoted apoptosis of TMZ-resistant GBM cells in a dose-dependent manner. Our results provide a major boost to the clinical trials with dacomitinib in GBM patients and a possible synergistic strategy was proposed for the combination of dacomitinib and TMZ in refractory GBM patients.

RNAi is a posttranscriptional gene silencing method triggered by siRNAs that has potential therapeutic applicability in cancer [55]. However, there are major limitations in the use of siRNAs as therapeutic tools, including their degradation by extracellular nucleases and poor cellular uptake [56]. Although numerous vehicles, such as cationic liposomes, viral vectors and nanoparticles, have been used to transport siRNAs, each method has disadvantages [57]. Therefore, delivering interfering RNAs to tumor cells in vivo remains challenging. In this study, a novel GBM-targeting nanoplatform, ANG-exo, was successfully constructed. A cholesterol-modified multi-siRNA, which simultaneously suppressed circCABIN1 and OLFML3 expression, was chosen as the chemosensitizing drug. Notably, Ang-multi-siRNA-exo exhibited favorable BBB permeability and tumor accumulation capacity in orthotopic GBM xenografts. In addition, Ang-multi-siRNA-exo sensitized GBM to TMZ in vivo, with low toxicity and might solve the current problems in RNAi-based therapeutics and propel the application of modified exosomes for cancer therapy.

## Conclusion

In summary, this study proposes a novel mechanism for drug resistance transmission in solid tumors. EIF4A3-induced circCABIN1 directly transmits the resistance phenotype to recipient GBM cells through exosomes by reprogramming the cancer stemness signature. Exosomal circCABIN1 competitively sponges miR-637 to block the suppressive effect of miR-637 on OLFML3 and then contributes to stemness reprogramming and TMZ resistance. We also provide evidence that engineered exosomes are a promising clinical tool for cancer prevention and therapy.

## Supplementary Information

**Additional file 1. **Additional materials. **Fig. S1** Related to Fig. 1. **A** Confirmation of the drug resistance of TMZ-resistant GBM02 cells in vivo. Left, IVIS detects bioluminescence signals. Right, quantification of bioluminescent imaging signal intensities. **B** Transmission electron micrograph of Res-exo and Sen-exo. **C** The effect of GW4869 and Rab27a/b siRNA on LN229 / LN229 TMZ resistant cells by CCK-8 assay. **D** Cells administrated Res-exo or Sen-exo were subjected to FACS to detected apoptosis. **E** Schematic image and statistical chart of processing of determination time of IC50 after Res-exo or Sen-exo treatment. **F** The effect of Res-exo or Res-exo plus Sen-exo in LN229 cells by CCK-8 assay. **Fig. S2** Related to Fig. 2. **A-B** Circus plot and heatmap presents the distribution and expression profiles of the detected and differentially expressed circRNAs on human chromosomes. **C** The existence of circCABIN1 was confirmed by RT-PCR and gel electrophoresis using convergent and divergent primers. **D** The level of circCABIN1 and linear CABIN1 treated with RNase R was detected by RT-PCR and gel electrophoresis. **E** Relative level of circCABIN1 and linear CABIN1 treated with RNase R was detected by qRT-PCR. **F** Relative level of circCABIN1 and linear CABIN1 treated with Actinomycin D was detected by qRT-PCR. **G** TMZ resistant cells were transfected with circCABIN1^KD^ and apoptosis was detected by caspase3/7 experiment. Scale bar, 10μm. **H** The expression of CABIN1 in different stages GBM and Kaplan-Meier analysis of OS in the high and low CABIN1 groups in public database**. Fig. S3** Related to Fig. 4.** A** GBM cells administrated with Sen-exo were subjected to FACS to detect the proportion of CD44^+^CD133^+^ cells. **B** The expression of circCABIN1 in the CD44^+^CD133^+^ or CD44^－^CD133^－^ cells was analyzed by qRT-PCR. **C** The expression of circCABIN1 in the tumorsphere and adherent from orthotopic GBM xenograft mice was analyzed by qRT-PCR. **D** Limiting dilution analysis assays detected the stem cell properties of cells transfected with shNC or circCABIN1^KD^. **Fig. S4** Related to Fig. 5. **A **The expression of miR-637 in the CD44^+^CD133^+^ or CD44^－^CD133^－^ cells was analyzed by qRT-PCR. **B** The expression of miR-637 in TMZ resistant and parental cells was analyzed by qRT-PCR. **C** Colony formation assay detected the effect of cells treated with control vector, miR-637 mimics alone or miR-637 mimics plus circCABIN1^OE^. **Fig. S5** Related to Fig. 6. **A** Kaplan-Meier analysis of OS in the high and low CITED4, ITGA10, GLDC and EIF4EBP3 groups in TCGA. **B** Kaplan-Meier analysis of OS in the high and low OLFML3 groups in CGGA, Gravendeel and Rembrandt database. **C** The expression of OLFML3 in glioma and matched normal tissue in public database. **D** KEGG pathway analysis demonstrated that regulating pluripotency of stem cells was involved and might be the downstream of OLFML3. **E** The correlation between OLFML3 and key stemness molecules according to CGGA database. **F** Colony formation assay detected the effect of cells treated with OLFML3 recombinant protein (0.5μg/mL). **G** The expression of OLFML3 in TMZ resistant and parental cells were analyzed by western blot. **Fig. S6** Related to Fig. 7. **A-B** Detection of circCABIN1 and OLFML3 knockdown efficiency by multi-siRNA. **C** The size distribution of ummod-exo and multi-siRNA-exo measured by NANO SIGHT. **D **Transmission electron micrograph of ummod-exo and multi-siRNA-exo. **E** The effect of ANG-multi-siNC-exo or ANG-multi-siRNA-exo plus TMZ treatment in cells. Left, apoptosis was detected by caspase3/7 experiment. Scale bar, 10μm. Right, cell viability was detected by CCK-8 assay. **F** The expression of OLFML3 in cells administrated with ANG-multi-siNC-exo or ANG-multi-siRNA-exo by western blot. **G** Clinical chemistry and hematology parameters for ANG-multi-siRNA-exo or Saline treated mice. **H** Body weight of BALB/c mice post-intravenous injection of ANG-multi-siRNA-exo or Saline. **I** Histological analyses of liver, heart, kidney, lung and spleen sections stained with H&E of BALB/c mice post-intravenous injection of ANG-multi-siRNA-exo or Saline for 12 days (one dose every other day).

## Data Availability

All data generated or analyzed during this study are included in this published article or available from the corresponding author on reasonable request.

## Abbreviations

GBM: Glioblastoma multiforme
TMZ: Temozolomide
BBB: Blood–brain barrier
circRNA: Circular RNA
lncRNA: Long noncoding RNA
miRNA: MicroRNA
exo: Exosome
multi-siRNA: Multiplex small interfering RNA
siRNA small: Interfering RNA
GFP: Green fluorescent protein
nSMase: Neutral sphingomyelinase-2
PDO: Patient-derived organoid
RT-PCR: Reverse transcription PCR
FISH: Fluorescence in situ hybridization
shRNA: Short hairpin RNA
EIF4A3: Eukaryotic initiation factor 4A-III
RBP: RNA-binding protein
ceRNA: Competing endogenous RNA
OLFML3: Olfactomedin-like 3
ANG: Angiopep-2
