# Supplementary material for: Exosome-transmitted circCABIN1 promotes temozolomide resistance in glioblastoma via sustaining ErbB downstream signaling
Source: J Nanobiotechnology. 2023 Feb 8;21:45. doi: 10.1186/s12951-023-01801-w (PMC9906870; doi:10.1186/s12951-023-01801-w)
Supplement: Supplementary file 1 — Additional file 1. Additional materials. Fig. S1 Related to Fig. 1. A Confirmation of the drug resistance of TMZ-resistant GBM02 cells in vivo. Left, IVIS detects bioluminescence signals. Right, quantification of bioluminescent imaging signal intensities. B Transmission electron micrograph of Res-exo and Sen-exo. C The effect of GW4869 and Rab27a/b siRNA on LN229 / LN229 TMZ resistant cells by CCK-8 assay. D Cells administrated Res-exo or Sen-exo were subjected to FACS to detected apoptosis. E Schematic image and statistical chart of processing of determination time of IC50 after Res-exo or Sen-exo treatment. F The effect of Res-exo or Res-exo plus Sen-exo in LN229 cells by CCK-8 assay. Fig. S2 Related to Fig. 2. A-B Circus plot and heatmap presents the distribution and expression profiles of the detected and differentially expressed circRNAs on human chromosomes. C The existence of circCABIN1 was confirmed by RT-PCR and gel electrophoresis using convergent and divergent primers. D The level of circCABIN1 and linear CABIN1 treated with RNase R was detected by RT-PCR and gel electrophoresis. E Relative level of circCABIN1 and linear CABIN1 treated with RNase R was detected by qRT-PCR. F Relative level of circCABIN1 and linear CABIN1 treated with Actinomycin D was detected by qRT-PCR. G TMZ resistant cells were transfected with circCABIN1KD and apoptosis was detected by caspase3/7 experiment. Scale bar, 10μm. H The expression of CABIN1 in different stages GBM and Kaplan-Meier analysis of OS in the high and low CABIN1 groups in public database. Fig. S3 Related to Fig. 4. A GBM cells administrated with Sen-exo were subjected to FACS to detect the proportion of CD44+CD133+ cells. B The expression of circCABIN1 in the CD44+CD133+ or CD44－CD133－ cells was analyzed by qRT-PCR. C The expression of circCABIN1 in the tumorsphere and adherent from orthotopic GBM xenograft mice was analyzed by qRT-PCR. D Limiting dilution analysis assays detected the stem cell properties of cell [file 12951_2023_1801_MOESM1_ESM.docx]

**Additional file 1**

**Figures**





**Fig. S1** Related to Fig. 1. **A** Confirmation of the drug resistance of TMZ-resistant GBM02 cells in vivo. Left, IVIS detects bioluminescence signals. Right, quantification of bioluminescent imaging signal intensities. **B** Transmission electron micrograph of Res-exo and Sen-exo. **C** The effect of GW4869 and Rab27a/b siRNA on LN229 / LN229 TMZ resistant cells by CCK-8 assay. **D** Cells administrated Res-exo or Sen-exo were subjected to FACS to detected apoptosis. **E** Schematic image and statistical chart of processing of determination time of IC50 after Res-exo or Sen-exo treatment. **F** The effect of Res-exo or Res-exo plus Sen-exo in LN229 cells by CCK-8 assay.





**Fig. S2** Related to Fig. 2. **A-B** Circus plot and heatmap presents the distribution and expression profiles of the detected and differentially expressed circRNAs on human chromosomes. **C** The existence of circCABIN1 was confirmed by RT-PCR and gel electrophoresis using convergent and divergent primers. **D** The level of circCABIN1 and linear CABIN1 treated with RNase R was detected by RT-PCR and gel electrophoresis. **E** Relative level of circCABIN1 and linear CABIN1 treated with RNase R was detected by qRT-PCR. **F** Relative level of circCABIN1 and linear CABIN1 treated with Actinomycin D was detected by qRT-PCR. **G** TMZ resistant cells were transfected with circCABIN1^KD^ and apoptosis was detected by caspase3/7 experiment. Scale bar, 10μm. **H** The expression of CABIN1 in different stages GBM and Kaplan-Meier analysis of OS in the high and low CABIN1 groups in public database.





**Fig. S3** Related to Fig. 4. **A** GBM cells administrated with Sen-exo were subjected to FACS to detect the proportion of CD44^+^CD133^+^ cells. **B** The expression of circCABIN1 in the CD44^+^CD133^+^ or CD44^－^CD133^－^ cells was analyzed by qRT-PCR. **C** The expression of circCABIN1 in the tumorsphere and adherent from orthotopic GBM xenograft mice was analyzed by qRT-PCR. **D** Limiting dilution analysis assays detected the stem cell properties of cells transfected with shNC or circCABIN1^KD^.





**Fig. S4** Related to Fig. 5. **A** The expression of miR-637 in the CD44^+^CD133^+^ or CD44^－^CD133^－^ cells was analyzed by qRT-PCR. **B** The expression of miR-637 in TMZ resistant and parental cells was analyzed by qRT-PCR. **C** Colony formation assay detected the effect of cells treated with control vector, miR-637 mimics alone or miR-637 mimics plus circCABIN1^OE^.





**Fig. S5** Related to Fig. 6. **A** Kaplan-Meier analysis of OS in the high and low CITED4, ITGA10, GLDC and EIF4EBP3 groups in TCGA. **B** Kaplan-Meier analysis of OS in the high and low OLFML3 groups in CGGA, Gravendeel and Rembrandt database. **C** The expression of OLFML3 in glioma and matched normal tissue in public database. **D** KEGG pathway analysis demonstrated that regulating pluripotency of stem cells was involved and might be the downstream of OLFML3. **E** The correlation between OLFML3 and key stemness molecules according to CGGA database. **F** Colony formation assay detected the effect of cells treated with OLFML3 recombinant protein (0.5μg/mL). **G** The expression of OLFML3 in TMZ resistant and parental cells were analyzed by western blot.





**Fig. S6** Related to Fig. 7. **A-B** Detection of circCABIN1 and OLFML3 knockdown efficiency by multi-siRNA. **C** The size distribution of ummod-exo and multi-siRNA-exo measured by NANO SIGHT. **D** Transmission electron micrograph of ummod-exo and multi-siRNA-exo. **E** The effect of ANG-multi-siNC-exo or ANG-multi-siRNA-exo plus TMZ treatment in cells. Left, apoptosis was detected by caspase3/7 experiment. Scale bar, 10μm. Right, cell viability was detected by CCK-8 assay. **F** The expression of OLFML3 in cells administrated with ANG-multi-siNC-exo or ANG-multi-siRNA-exo by western blot. **G** Clinical chemistry and hematology parameters for ANG-multi-siRNA-exo or Saline treated mice. **H** Body weight of BALB/c mice post-intravenous injection of ANG-multi-siRNA-exo or Saline. **I** Histological analyses of liver, heart, kidney, lung and spleen sections stained with H&E of BALB/c mice post-intravenous injection of ANG-multi-siRNA-exo or Saline for 12 days (one dose every other day).

**Key resources table**

| REAGENT or RESOURCE | SOURCE | IDENTIFIER |
| --- | --- | --- |
| Antibodies |  |  |
| Anti-OLFML3 | ThermoFisher Scientific | Cat# PA5-31581 |
| Anti-GAPDH | Cell Signaling Technology | Cat# 5174 |
| Anti-EIF4A3 | Proteintech | Cat# 17504-1-AP |
| Anti-CD44 | Cell Signaling Technology | Cat# 5640 |
| Anti-Nestin | Cell Signaling Technology | Cat#73349 |
| Anti-CD133 | Abcam | Cat# ab284389 |
| Anti-SOX2 | Abcam | Cat# ab92494 |
| Anti-OCT4 | Abcam | Cat#ab181557 |
| Anti-ALDH1A3 | Abcam | Cat#ab129815 |
| Anti-AGO2 | Abcam | Cat#ab186733 |
| Anti-Ki67 | Abcam | Cat# ab15580 |
| Anti-caspase 3 | Abcam | Cat# ab32351 |
| CoraLite®488-Phalloidin (green) | Proteintech | PF00001 |
| CoraLite488-conjugated Goat Anti-Mouse IgG(H+L) | Proteintech | Cat#SA00013-1 |
| CoraLite488-conjugated Goat Anti-Rabbit IgG(H+L) | Proteintech | Cat#SA00013-2 |
| Cy3-conjugated Goat Anti-Mouse IgG(H+L) | Proteintech | Cat#SA00009-1 |
| Cy3-conjugated Goat Anti-Rabbit IgG(H+L) | Proteintech | Cat#SA00009-2 |
| Anti-Rabbit IgG, HRP-linked Antibody | Cell Signaling Technology | Cat#7074 |
| Anti-Mouse IgG, HRP-linked Antibody | Cell Signaling Technology | Cat#7076 |
| Goat Anti-Rabbit IgG H&L (HRP) | Abcam | Cat# ab6721 |
| Goat Anti-Mouse IgG H&L (HRP) | Abcam | Cat# ab6789 |
| Chemicals, Peptides, and Recombinant Proteins |  |  |
| DMSO | Sigma-Aldrich | Cat# D8418 |
| 0.25 Trypsin solution (without EDTA) | Servicebio | Cat#G4002 |
| 0.25 Trypsin solution (containing EDTA) | Servicebio | Cat#G4004 |
| Matrigengel | ABW | Cat#082721 |
| D-Luciferin potassium salt | Abcam | Cat#ab143655 |
| Lipofectamine ^®^ 3000 | Invitrogen | Cat#L3000001 |
| PrimeScript^TM^ RT Master Mix | Takara | Cat#RR036A |
| PrimeScript^TM^ RT Master Mix | Takara | Cat# RR037A |
| Fetal bovine serum | Gibco | Cat# 10099141C |
| Fast SYBR^®^ Green Master Mix | ThermoFisher Scientific | Cat#4385612 |
| Penicillin-Streptomycin | NCM | Cat#C100C5 |
| OLFML3 Fusion Protein | Proteintech | Cat#Ag8655 |
| Dulbecco’s modified Eagle’s medium | Gibco | Cat#11995500 |
| Opti-MEM | Gibco | Cat#31985070 |
| TRIzol | 1559606 | Cat#15596026 |
| Western&IP lysis buffer | NCM | Cat#P70100 |
| Exosome Isolation Reagent (for cell culture media) | RiboBio | Cat#C10130-2 |
| Temozolomide | TargetMol | Cat#85622-93-1 |
| Dacomitnib | Selleck | Cat#S2727 |
| Critical Commercial Assays |  |  |
| PE Annexin V Apoptosis Detection Kit I | BD Biosciences | Cat#559763 |
| PARIS™ | ThermoFisher Scientific | Cat#AM1921 |
| Pierce™ Rapid Gold BCA Protein Assay Kit | ThermoFisher Scientific | Cat#A53225 |
| Pierce™ RNA 3’ End Desthiobiotinylation Kit | ThermoFisher Scientific | Cat#20163 |
| Pierce™ Magnetic RNA-protein Pull-Down Kit | ThermoFisher Scientific | Cat#20164Y |
| Magna RIP™ Kit | EMD Millipore | Cat#MAGNARIP01 |
| Apo-ONE® Homogeneous Caspase-3/7 Assay Kit | Promega | Cat#G7790 |
| M5 Protein Silver Stain Kit | Mei5bio | Cat#MF329-01 |
| Human and Mouse AKT Pathway Phosphorylation Array C1 | RayBio | Cat# AAH-AKT-1-4 |
| Fluorescent In Situ Hybridization Kit | RiboBio | Cat#C10910 |
| Cell Counting Kit 8 | Abcam | Cat#ab228554 |
| Experimental Models: Cell Lines |  |  |
| LN229 | ATCC | Bio-105840a |
| GBM02 | This study | N/A |
| GBM04 | This study | N/A |
| GBM14 | This study | N/A |
| Experimental Models: |  |  |
| Balb/c nude mice | Gempharmatech | Cat#D000521 |
| Balb/c mice | Laboratory Animal Center, Fourth Military Medical University | N/A |
| Software and Algorithms |  |  |
| ImageJ | https://imagej.nih.gov/ij/ |  |
| GraphPad Prism 7 | GraphPad Software |  |
| R package | http://cran.us.r-project.org |  |
| FlowJo 10.0 | TreeStar |  |
| Deposited Data |  |  |
| CGGA | http://www.cgga.org.cn/ |  |
| Brainbase | https://ngdc.cncb.ac.cn |  |
| Gliovis | http://gliovis.bioinfo.cnio.es/ |  |
| miRanda | http://www.microrna.org |  |
| RNAhybrid | https://bibiserv.cebitec.uni-bielefeld.de/ |  |
| Circinteractome | https://circinteractome.nia.nih.gov/ |  |
| miRwalk | http://mirwalk.umm.uni-heidelberg.de/ |  |
| Targetscan | https://www.targetscan.org/ |  |

[**Ncleotide**](javascript:;) [**sequence**](javascript:;)

| Gene | siRNA | sequence |
| --- | --- | --- |
| human-EIF4A3 | siRNA#1 | sense(5'-3') CCACAAUGUUGACAAACUAAA |
|  |  | antisense(5'-3') UAGUUUGUCAACAUUGUGGUA |
|  | siRNA#2 | sense(5'-3') GGAUCAUGACUUACCACAAUG |
|  |  | antisense(5'-3') UUGUGGUAAGUCAUGAUCCUG |
| human-FUS | siRNA#1 | sense(5'-3') GGCUAUGGAACUCAGUCAACU |
|  |  | antisense(5'-3') UUGACUGAGUUCCAUAGCCUG |
|  | siRNA#2 | sense(5'-3') GGACAGCAGCAAAGCUAUAAU |
|  |  | antisense(5'-3') UAUAGCUUUGCUGCUGUCCAU |
| human- PTBP1 | siRNA#1 | sense(5'-3') GCAAGAAGUUCAAAGGUGACA |
|  |  | antisense(5'-3') UCACCUUUGAACUUCUUGCUG |
|  | siRNA#2 | sense(5'-3') GCCUCAACGUCAAGUACAACA |
|  |  | antisense(5'-3') UUGUACUUGACGUUGAGGCUG |
| human-circCABIN1 | siRNA | sense(5'-3') CAAUUUCUUCAACGUGUACTT |
|  |  | antisense(5'-3') GUACACGUUGAAGAAAUUGTT |
| human-OLFML3 | siRNA#1 | sense(5'-3') GGAAUGAGAAGUACGAUAUTT |
|  |  | antisense(5'-3') AUAUCGUACUUCUCAUUCCTT |
|  | siRNA#2 | sense(5'-3') CAGAGAAGAUCUACGUGUUTT |
|  |  | antisense(5'-3') AACACGUAGAUCUUCUCUGTT |
|  | siRNA#3 | sense(5'-3') GAGAACACUUUGCAGCUAATT |
|  |  | antisense(5'-3') UUAGCUGCAAAGUGUUCUCTT |
| hsa-miR637 | MIMICS | sense(5'-3') ACUGGGGGCUUUCGGGCUCUGCGU |
|  |  | antisense(5'-3') GCAGAGCCCGAAAGCCCCCAGUUU |

**Primer sequence**

| human-circCABIN1 | FORWARD——CGCTTCCCCCAGCACTATAA |
| --- | --- |
|  | REVERSE——GATGGAAGCATGGAGCCGAA |
| hsa-miR637 | FORWARD——TGGGGGCTTTCGGGCT |
|  | REVERSE——AGTGCAGGGTCCGAGGTATT |
| human-U6 | FORWARD——TGCTTCGGCAGCACATATAC |
|  | REVERSE——TCACGAATTTGCGTGTCATC |
| human-GAPDH | FORWARD——AGAAGGCTGGGGCTCATTTG |
|  | REVERSE——AGGGGCCATCCACAGTCTTC |
| RIP-a | FORWARD——TCCAGAGTTCAGTGGGGAGA |
|  | REVERSE——CTTCAGCTCCGTTCACTGGG |
| RIP-b | FORWARD——GTGAACGGAGCTGAAGGGTT |
|  | REVERSE——CTGCCACACACTGCTTGTTT |
| RIP-c | FORWARD——GCTAACAAAAACAAGCAGTGTGTG |
|  | REVERSE——AGCCTTCCCAGTTGCTAAGAT |
| RIP-d | FORWARD——CAGGCAACGCCGTGTACTT |
|  | REVERSE——TCCTGCGTTGAGCTAGAGTC |
| RIP-e | FORWARD——TGAGTACTTTGCCTTGTTGATTTCC |
|  | REVERSE——TTGAGGCCCTGGGAACACC |
| RIP-f | FORWARD——CTCAAGTTTGCGGCATCCTC |
|  | REVERSE——TGACTTGCCAGAAGTCGCTC |
| RIP-g | FORWARD——TCCAAAGCCCCAGGTATTCTG |
|  | REVERSE——TCTCCTACGTGCTTGAGACC |
| hsa-OLFML3 | FORWARD——ACGTTCCTTCTACTCTGGCAC |
|  | REVERSE——TCTAAAGCAGCTAGTCGGCG |
| hsa-miR892b | FORWARD——GCGCACTGGCTCCTTTCTG |
|  | REVERSE——AGTGCAGGGTCCGAGGTATT |
| hsa-miR671-5p | FORWARD——AGGAAGCCCTGGAGGGG |
|  | REVERSE——AGTGCAGGGTCCGAGGTATT |
| hsa-miR665 | FORWARD——CGCGACCAGGAGGCTGAG |
|  | REVERSE——AGTGCAGGGTCCGAGGTATT |
| hsa-miR663b | FORWARD——GGTGGCCCGGCCGTGC |
|  | REVERSE——AGTGCAGGGTCCGAGGTATT |
| hsa-miR331-3p | FORWARD——CGGCCCCTGGGCCTATC |
|  | REVERSE——AGTGCAGGGTCCGAGGTATT |
| hsa-miR1225-3p | FORWARD——TGAGCCCCTGTGCCGC |
|  | REVERSE——AGTGCAGGGTCCGAGGTATT |
| hsa-miR1184 | FORWARD——CGCCTGCAGCGACTTGATG |
|  | REVERSE——AGTGCAGGGTCCGAGGTATT |
